# Occurrence and Distribution of Persistent Organic Pollutants (POPs) from Sele River, Southern Italy: Analysis of Polychlorinated Biphenyls and Organochlorine Pesticides in a Water–Sediment System

**DOI:** 10.3390/toxics10110662

**Published:** 2022-11-04

**Authors:** Elvira De Rosa, Paolo Montuori, Maria Triassi, Armando Masucci, Antonio Nardone

**Affiliations:** Department of Public Health, University “Federico II”, Via Sergio Pansini nº 5, 80131 Naples, Italy

**Keywords:** persistent organic pollutants, Sele river, toxicity equivalent, risk assessment, Principal Component Analysis

## Abstract

The concentrations, possible sources, and ecological risk of polychlorinated biphenyls (PCBs) and organochlorine pesticides (OCPs) were studied by analyzing water column (DP), suspended particulate matter (SPM) and sediment samples from 10 sites on the Sele River. Total PCBs concentration ranged from 2.94 to 54.4 ng/L and 5.01 to 79.3 ng/g in the seawater and sediment samples, with OCPs concentration in the range of 0.51 to 8.76 ng/L and 0.50 to 10.2 ng/g, respectively. Pollutants loads in the seaside were measured in approximately 89.7 kg/year (73.2 kg/year of PCBs and 16.5 kg/year of OCPs), indicating that the watercourse could be an important cause of contamination to the Tyrrhenian Sea. Statistical analysis indicates that all polychlorinated biphenyls analytes are more probable to derive from surface runoff than an atmospheric deposition. The results explain that higher concentrations of these pollutants were built in sediment samples rather than in the other two phases, which are evidence of historical loads of PCBs and OCPs contaminants. The Sediment Quality Guidelines (SQGs), the Ecological Risk Index (ERI) and the Risk Quotient (RQ) show that the Sele river and its estuary would reputedly be a zone possibly at risk.

## 1. Introduction

The importance of riverine ecosystems for human living has attracted the interest of authorities and researchers, especially after the development of cities and the increase in industrial and agricultural activities, which have released significant amounts of contaminants into these ecosystems [1,2,3]. Among these, the persistent organic pollutants (POPs) such as Polychlorinated biphenyls (PCBs) and Organochlorine pesticides (OCPs) [4], have raised concern due to their physico-chemical properties and high toxicity [5].

POPs are a set of toxic chemicals that are persistent in the environment and able to last for several years before breaking down. Several were concluded regionally and globally to develop better risk management so as to reduce the impact of these toxic substances on humans’ health and the environment [6]. Among these treaties, the Stockholm Convention on POPs is the most important. Accordingly, it has been necessary to introduce a set of rules for the forbidden and restricted worldwide use of POPs that are harmful to human health and the environment, because these are very stable compounds that resist photolytic, biological and chemical degradation and that thus persist in the environment with long half-lives [7,8]. These compounds can be transferred from air to surface soil and water by dry and wet deposition, from soil to aquatic bodies by rainfall runoff, and from soil and aquatic bodies back to air by volatilization. Due to the long-range atmospheric transport, they have been found in most areas of the world [9,10]. They greatly affect the quality of environmental ecosystems and human health.

PCBs are man-made organic compounds composed of a biphenyl with different numbers of chlorine atoms replaced with two six-carbon benzene rings [11]. They are composed of more than 200 individual chemical compounds produced by industrial mixtures via introducing elementary chlorine into biphenyl. Therefore, the primary source of PCBs is industrial production, including industrial wastewaters and slag discharged into the receiving environment. PCBs could have 10 homologs and 209 distinct congeners counting on the number and location of chlorine atoms. Given their property of low electrical conductivity and high resistance to heat and thermal degradation, PCBs are applied as heat exchange fluids in transformers and capacitors. Furthermore, PCBs were ideal additives in paints, dyed paper and plastics [12].

OCPs have been extensively applied in agriculture worldwide for several decades and they mainly originate from improperly treated industrial wastewaters originating from pesticide manufacturing plants. Different species of OCPs, including hexachlorocyclohexanes (HCHs) and dichlorodiphenyltrichloroethanes (DDTs), are still extensively present in water, sediments, atmosphere, fish and even food, due to their persistence, even though the production and application of these contaminants were banned in evolved countries in the 1970s and 1980s [13,14]. Because of their high refractiveness and hydrophobicity, most OCPs firmly adhere to the surface of suspended particles and eventually to sediments at the bottom of water bodies when entering the water environment. They can be subsequently released into the water column under certain conditions such as water turbulence, posing a serious threat to aquatic organisms and human health [15,16,17].

Many studies have confirmed that the marine environment appears to be one of the primary locations for the accumulation of PCBs and OCPs [18,19,20].

This study investigates the concentrations of PCBs and OCPs found from the Sele river, one of the main rivers of the Campania plain. Campania is one of the most populated regions of Italy, in which are developed numerous industrial activity and rich agricultural practices such as livestock farming (buffalo farms); the large-scale production of vegetables and fruits feeds the local food industry. These activities include a vast use of pesticides and fertilizers, which can damage water quality [21,22].

Hence, this study is intended to evaluate the concentrations of PCBs and OCPs from the Sele river estuary, southern Italy, and their environmental impact on the Mediterranean Sea. In particular, this paper aims to (i) estimate the PCBs and OCPs levels from the Sele river estuary; (ii) evaluate their distribution between the phases analyzed; (iii) define their spatial distribution and temporal trends in the study area; (iv) assess the potential environmental impact of PCBs and OCPs from the Sele river on the Mediterranean Sea. To the best of our knowledge, there are no previous studies that have evaluated the loads of PCBs and OCPs from the Sele river and the environmental impact on the Mediterranean Sea.

## 2. Materials and Methods

### 2.1. Study Area

**Figure 1:**
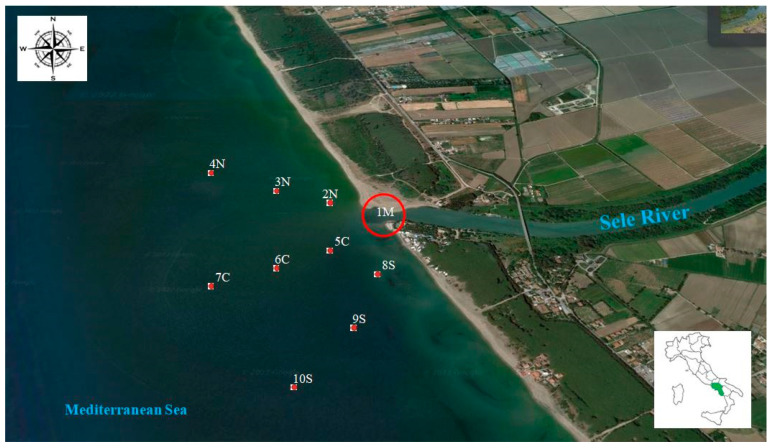
Study area in the Mediterranean Central Sea: solid dots show sampling stations from the Sele river and estuary, southern Italy.

The Sele river is the second river of the Campania region in the South of Italy, after the Volturno river, and it is a tributary of the Tyrrhenian Sea. It is one of the most important watercourses of the region with a drainage basin of 3235 km^2^, a length of 64 km and an annual mean flow rate of 69 m^3^/s (Figure 1) [21,23]. The basin is located on the western (i.e., Tyrrhenian) side of southern Italy and includes a large alluvial plain. The plain has a triangular surface area of about 400 km^2^ and it is flanked versus the sea by a straight sandy coast between the towns of Salerno and Agropoli. In the Campania region (CP), the city of Salerno is amongst the most tourist-oriented areas around the Mediterranean Sea; furthermore, it has one of the largest transportation networks in south Italy, including railway, highway and various road connections into and around the region. The Sele plain is characterized by agriculture and agro-industries that still provides the major economic income and, from an environmental point of view, the stream network system in the Sele plain is responsible for carrying fertilizers and related products into the Mediterranean Sea. Instead, in the last decade, another source of pollution has been represented by a large number of illegal waste dumps, uncontrolled burning sites (especially in the north of Campania) and industrial wastes from manufacturing enterprises operating in the textile and leather goods sector, which contribute to an increase in the concentrations of the main pollutants [24,25].

The Sele river basin is characterized by a Mediterranean climate with a particularly dry climate in summer and mild temperatures in winter. The sea contributes to determining the climate, which is warm temperate, with modest daily and annual temperature ranges (less than 21 °C); in fact, the sea maintains the summer heat, accumulating and then releasing it during the winter. The dry summers and rainy winters are a typical characteristic of the Mediterranean climate [26,27].

### 2.2. Sample Collection

To assess temporal trends of pollutants, between 2020 and 2021, four sampling campaigns were conducted in the summer, autumn, winter and spring from 10 sampling points along the Sele river: the first sampling point was the Sele mouth and the other nine were at diverse distances from the mouth, i.e., 500 m, 1000 m and 1500 m to the north, south and west (Table 1). Three aliquots were sampled at each chosen point and for each season. Once collected, the samples were carried out to the laboratory and analyzed in triplicate, in order to assess the repeatability of the method. For any locations 2.5 L of water (approximately a depth of 0–50 cm from the sampling points) were collected from the surface layer with amber bottles using a portable water collector. All water samples were sent to the laboratory and placed in a 4 °C refrigerator. Sediment samples were obtained at a depth of 0–5 cm in a 0.04 m^2^ range area with a Van Veen Grab sampler, and the overall weight of the sediment samples was not less than 500 g. The samples were quickly wrapped in polyethylene bags, shipped to the laboratory and placed in a refrigerator at −20 °C.

### 2.3. Sample Processing and Chemical Analysis

The method used for extraction and analytical determination has been published previously [28]. Briefly, water samples were filtered through a previously kiln-fired (400 °C overnight) GF/F glass fiber filter (47 mm × 0.7 µm; Whatman, Maidstone, UK). Filters (suspended particulate matter, SPM) were kept in the dark at −20 °C until analysis. Dissolved phases (fraction of contaminants passing through the filter) were kept in the dark at 4 °C and extracted within the same day of sampling (3–6 h from sampling). Filters were fortified with 2 ng of PCB #65 and PCB #166 as a recovery standard, respectively. After, they were extracted three times by sonication and concentrated to 0.5 mL [29]. The dissolved phase (DP) was fortified with PCB #65 and PCB #166 as a recovery standard, in order to obtain a final concentration of 5 ng L^−1^. Two liters of sample (DP) were preconcentrated and analyzed using SPE for solid phase extraction; subsequently, they were eluted and concentrated at 0.5 mL.

Sediments were oven desiccated at 60 °C and sifted at 250 μm. A samples rate was fortified with the same surrogate standards used previously, extracted three times and concentrated as the water samples [29]. In each sample analyzed of DP, SPM and sediment, the amount of the following 32 chosen PCBs were quantified (PCBs 8, 28, 37, 44, 49, 52, 60, 66, 70, 74, 77, 82, 87, 99, 101, 105, 114, 118, 126, 128, 138, 153, 156, 158, 166, 169, 170, 179, 180, 183, 187 and 189) (C-SCA-06 PCB Congeners Mix #6; AccuStandard, Inc., New Haven, CT 06513, USA). Instead, the mixed OCPs standard solution included: aldrin, α-BHC, βBHC, δ-BHC, γ-BHC (lindane), p,p′-DDD, p,p′-DDE, p,p′-DDT, dieldrin, endosulfan I, endosulfan II, endosulfan sulfate, endrin, endrin aldehyde, heptachlor, heptachlor epoxide (isomer B) and methoxychlor (M-8080 Organochlorine Pesticides; AccuStandard, Inc., CT 06513, USA). Analysis of sample extracts and standards was performed using a GC17A Shimadzu (Kyoto, Japan), equipped with an electron capture detector (ECD) and an AOC-20i Shimadzu (Kyoto, Japan) autosampler. Identification of the compounds was achieved by comparing the retention times of the samples with those of the individual PCBs, while quantitative analysis was based on multilevel calibration curves. To confirm the presence of OCPs, GC–MS using a GC–MS 2010 Plus Shimadzu (Kyoto, Japan) was used, working in the electron impact mode and operating at 70 eV.

The mass spectrometer was operated in Single-Ion Monitoring (SIM) mode with the molecular ions of the studied pollutants. PCBs and OCPs are quantified using the response factors of internal standards.

### 2.4. Quality Assurance and Quality Control

All results were subject to precise quality control process. For each set of 10 samples, a procedural blank and a spiked sample consisting of all reagents were used to check interferences and cross-contaminations. Surrogate standards in DP, SPM and SED samples were analyzed carefully. The mean recovery of a surrogate for the DP sample was 80.5 ± 8.2%, for SPM samples was 79.3 ± 6.2%, and for sediment samples was 83.7 ± 3.1%. Spiked samples in each set of 10 samples were analyzed with mean recoveries ranging from 78.8 to 102.7%. Each extract was evaluated in two copies, in addition, the errors involved in sampling were assessed by carrying out triplicate sampling of water and sediment at the same site and the analysis of sample extracts. Results showed good reproducibility of the sampling process.

The Metod Detection Limit (MDL) was calculated as the average blank values plus three times the standard deviation and it ranged from 0.006 to 0.100 ng L^−1^ in the dissolved phase and in the particulate phase and ranged from 0.0005 to 0.0050 ng g^−1^ in the sediment. IDL was calculated as three times the noise in a blank sample chromatogram. If the amount of any compound in a sample was under its MDL/IDL, this analyte was reputed as not detected in the sample (under the limit of detection, <LOD). Data obtained for PCBs and OCPs were rectified for surrogate recoveries.

### 2.5. Analysis and Contaminants Load

All statistical analyses were performed with the SPSS 22.0 statistical package (IBM-SPSS Inc., Chicago, IL, USA). The significance level was *p* < 0.05 unless otherwise stated.

According to the UNEP guidelines [30], the method to evaluate the annual pollutants loads has been used (F_annual_): The mean of the total concentrations was multiplied by the annual average flow rate (m^3^/year) of the Sele river for each sampling event and corrected by the total water load for the sampling period. The average flow considered is 69 m³/s and this information was found in the database of the Autorità di Bacino Distrettuale dell’Appennino Meridionale Sede Basilicata.

Principal Component Analysis (PCA) is a statistical process that purposes an orthogonal transformation to change a group of observations of potentially associated variables into a group of values of linearly uncorrelated variables called principal components. It is one of the oldest and most widely technique used. It reduces the dimensionality of a dataset, while preserving as much variability as possible [31]. In this study, PCA was performed to determine the possible sources of PCBs.

### 2.6. Toxicity and Dioxin-like PCBs

Dioxin-like PCBs (dl-PCBs) are compounds containing four to eight chlorine atoms. They are very toxic contaminants, bioaccumulative and pose a major health risk due to certain molecular characteristics. In fact, dl-PCBs have a comparable chemical conformation to dioxins and furans. For the combined risk assessment of these substances, the toxic equivalent (TEQ) concentrations for dioxin-like PCBs were calculated according to toxic equivalency factors (TEFs) adopted by the World Health Organization (WHO) in 2005 [32]. TEFs are a fundamental element of TEQ and have developed in the last few decades for dioxins/dioxin-like compounds.

TEF values used in this study are indicated by WHO 2005 for human and mammals [32]: 0.0001 for PCB 77; 0.0003 for PCB 81; 0.00003 for PCB 105, 114, 118, 123, 156, 157, 167 and 189; 0.03 for PCB 169 and 0.1 for PCB 126.

The maximum tolerable value established by US EPA is 0.7 pg WHO-TEQ/kg body weight, and the Equation (1) used to calculate the TEQ is the following:

ΣTEQ = ΣC_i_ × TEF_i_ (1)

C_i_ represents the amount of dl-PCBs (expressed in ng/g). In this study, the TEQ values were calculated in sediment samples to evaluate the presence of humans and environmental risks.

### 2.7. Risk Assessment

Sediment quality guidelines (SQGs) are generally employed as the effective tool for the estimation of ecological pollution of PCBs in the sediments samples, and have been used in many applications, including monitoring programs, ecological risk assessments and preventing additional pollution.

There are two set of SQGs identified as: (ERL) effect range low and (ERM) effect range median, which evaluate the probably negative effects on organisms concerning individual PCBs as well as the cumulative toxic effects due to the sum of total PCBs [33]; (TEL) threshold effect level and (PEL) probable effect level, which constitute the chemical amount under which the probability of toxicity and other effect are rare [28]. To evaluate the ecological risk related to PCBs and OCPs in the water environment, two indices have been estimated: The Ecological Risk Index (ERI) suggested by Hakanson [34], to evaluate the level of PCBs contamination in the watercourse environment; and Risk Quotient (RQ) method [35], for OCPs pollution. The ERI can be calculated using the following equations:

RI = ∑ *E^i^_r_* (2)

*E^i^_r_ = T^i^_r_ C^i^_f_* (3)

*C^i^_f_ = C^i^*_0_/*C^i^* (4)

where ERI is the sum of potential ecological risk for all trace PCBs in the sediments, ERI was equal to *E^i^_r_*, *E^i^_r_* and *T^i^_r_* are the toxicity coefficient and individual potential ecological risk for PCBs, which for these pollutants was equal to 40, in line with the standardization elaborated by Hakanson [34]. *C^i^_f_* was the contamination factor, *C^i^*_0_ was the PCBs amount in the sediment and *C^i^_n_* was an established value equal to 10 µg/kg. The interpretation and significance of ERI is given as follows: low potential ecological risk, ERI < 40; moderate potential ecological risk, ERI = 40–79; considerable potential ecological risk, ERI = 80–159; high potential ecological risk, ERI =160–319; and very high potential ecological risk, ERI > 320 [34]. Regarding OCPs, the risk quotient (RQ) was conducted via calculation of RQ using Equation (5):

RQ = C/PNEC (5)

where C was the concentration and PNEC was the predicted no-effect concentrations for particular OCPs. The PNEC results were procured from the ECOTOX database [36]. When RQ < 0.01, the OCP has a very low risk to aquatic organisms, and when 0.01 ≤ RQ < 0.1, the ecological risk level is low. When 0.1 ≤ RQ < 1, the OCP has a moderate risk to aquatic organisms. When 1 ≤ RQ < 10, the OCP has a high risk to aquatic organisms, and when RQ ≥ 10, the ecological risk level is very high [37,38].

## 3. Results and Discussions

### 3.1. PCBs Distribution in DP, SPM and Sediment Samples

**Figure 2:**
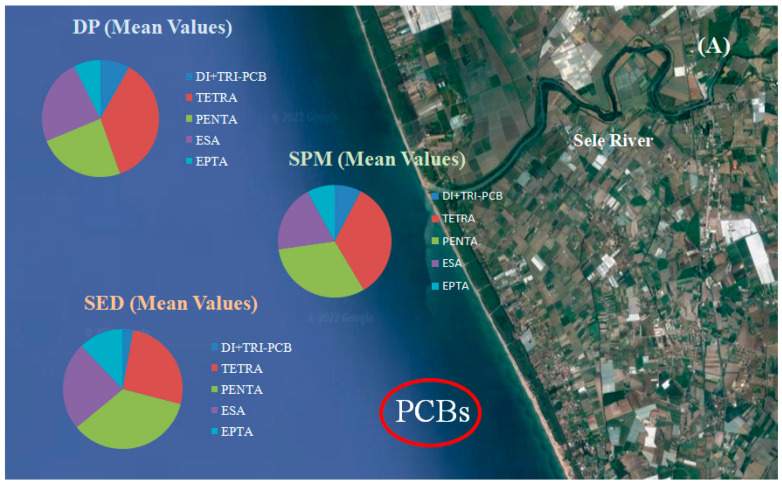

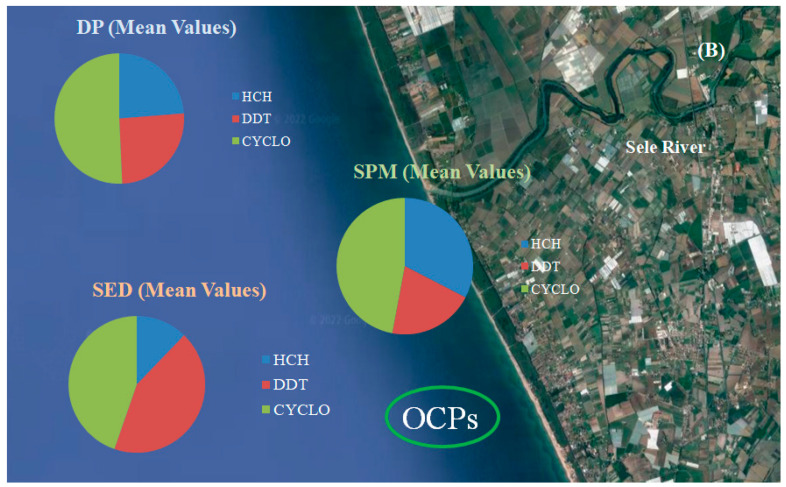
Mean concentrations of PCBs (**A**) and OCPs (**B**) in the water samples (DP), suspended particulate matter (SPM) and sediment (SED) from the Sele River, southern Italy.

PCBs were identified in all sampling sites. This result shows that PCBs are extensively spread in the study area. The sum of amounts of PCBs, as demonstrated in (Table 1 and Table S8), found in DP, extended from 1.98 ng L^−1^ (site 9) to 12.1 ng L^−1^ (site 1) with a mean value of 6.30 ± 2.10 ng L^−1^. In Tables S1–S3 (percentage values), the data show that, as reported in (Figure 2a), the main PCBs detected in collected samples were tetra, penta and hexa-CBs, suggesting an average over 82% of ΣPCBs. The abundant presence of this class of PCBs is probably due to the fact that these compounds have stronger hydrophilicity than PCBs, with more chlorine atom substitutions [39]; in fact, when the number of chlorine atoms increases, the solubility decreases [40,41]. In DP samples, hepta-CB were present only for 9% of total PCBs.

In the SPM phase, the PCBs concentrations varied from 0.35 ng L^−1^ (36.4 ng g^−1^) in site 8 to 35.1 ng L^−1^ (1895.3 ng g^−1^) in site 1 on dry weight (Table 1 and Table S9).

The PCBs most present are those with more chlorine atoms; in fact, in this phase, there is an increase in the percentage of hepta PCBs compared to the DP. This event can be explained through the chemical properties of the higher chlorinated PCBs, which are low hydrophilic and therefore, tend to bind more with the particulate (Figure 2a).

Regarding the sediment samples, the total PCBs values ranged from 5.0 ng g^−1^ (site 9) to 79.3 ng g^−1^ (site 1) (Table 1 and Table S10). Data show that the amount of hepta-PCBs increased to 10%. Moreover, the amount of di- + tri-PCBs decreased in sediments samples compared to SPM and DP samples. It can therefore be said that the percentage of highly chlorinated PCBs in the sediments samples was higher than that in the DP and SPM phases, and the percentage of less chlorinated PCBs was lower than that in the DP and SPM phases; furthermore, in the sediment have been found the highest concentrations of PCBs. The characteristic of PCBs depends on the degree of chlorination, i.e., the higher the degree of chlorination, the lower the water solubility and vapor pressure [39]. In the Sele river, sediments turn out to be a sink for these contaminants and are a measurement of their amount during the years [42,43,44]. PCBs being hydrophobic organic compounds, they are characterized by extraordinary stability, high toxicity, extremely high long-range atmospheric transportability [45,46]. In the aquatic environment, PCBs are removed from the water column and adsorbed onto suspended particulate matter; they can subsequently bio-accumulate in sediment and thereby, transfer to higher trophic levels through the food chain. Due to their persistent and hydrophobic nature, the fate and transport of PCBs in a water environment are highly affected by their adsorption behavior on the sediment [47,48]. Many factors influence the adsorption behavior of PCBs. In this study, among them, pH, temperature and salinity were considered. Salinity, for example, can alter the water solubility of hydrophobic compounds and the physicochemical properties of sediment, through which it influences the adsorption capacity of hydrophobic compounds on the sediment. Table S4 shows the data of the factors that may have contributed to a higher concentration of PCBs in the sediment and may have influenced the distribution of these contaminants analysed in this study characterized by a predominantly mineral sediment.

### 3.2. OCPs Distribution in DP, SPM and Sediment Samples

Data showed that samples raised from the Sele river included rests of HCH (sum of a-HCH, b-HCH, g-HCH, and d-HCH), DDT (p,p’-DDE, p,p’-DDD, p,p’-DDT isomers and methoxychlor) and cyclodienes (aldrin, dieldrin, endosulfan I, endosulfan II, endosulfan sulphate, endrin, heptachlor and heptachlor epoxide). In Table 2 and Table S11 were reported the results of the DP, SPM and sediment sample analyses. In the DP phase, the total concentrations varied from 0.36 ng L^−1^ (site 9) to 5.71 ng L^−1^ (site 1) (mean value of 1.22 ± 0.23 ng L^−1^). Particularly, as indicated in Figure 2b and in Tables S5–S7 (percentage values), they varied from ND to 0.75 ng L^−1^ for HCH, from ND to 1.0 ng L^−1^ for DDT and its degradates, and from ND to 3.20 ng L^−1^ for cyclodienes. In SPM, the amounts acquired for total OCPs extended from 0.05 ng L^−1^ (65.3 ng g^−1^ dw) in site 9 to 4.82 ng L^−1^ (201.4 ng g^−1^ dw) in site 1 (Table 2 and Table S12). The HCHs extended from ND to 0.89 ng L^−1^, the DDTs from ND to 0.96 ng L^−1^, and the cyclodienes from ND to 2.62 ng L^−1^, as shown in Figure 2b. In sediment samples, instead, the total OCPs concentration (Table 2 and Table S13) extended from 1.1 ng g^−1^ (site 9) to 15.0 ng g^−1^ (site 1). The HCHs ranged from 0.10 to 1.24 ng g^−1^, the DDTs from 0.10 to 6.12 and the cyclodienes from 0.15 to 3.10 ng g^−1^ (Figure 2b). The results show that in the Sele river, a higher percentage of cyclodienes and DDT was found compared to HCH; in fact, the results of the ratio indicate that the DDTs/cyclodienes ratio was <1 at most sites (mean, 0.70), such as the HCHs/DDTs and HCHs/cyclodienes ratios (means, 0.40 and 0.20, respectively). The dominant HCH was b-HCH (1.90 ± 1.00), followed by a-HCH (1.65 ± 0.80). This pesticide had a lower solubility in water, and dissolved organic matter can assimilate on this compound, which may raise the amount in water. The ratio of b-HCH in the HCHs was high and indicates that these contaminants maybe represent a historical input rather than a fresh input [49]. A similar trend for b-HCH has also been reported by Dong et al. [50] and Salem et al. [51].

**Figure 3:**
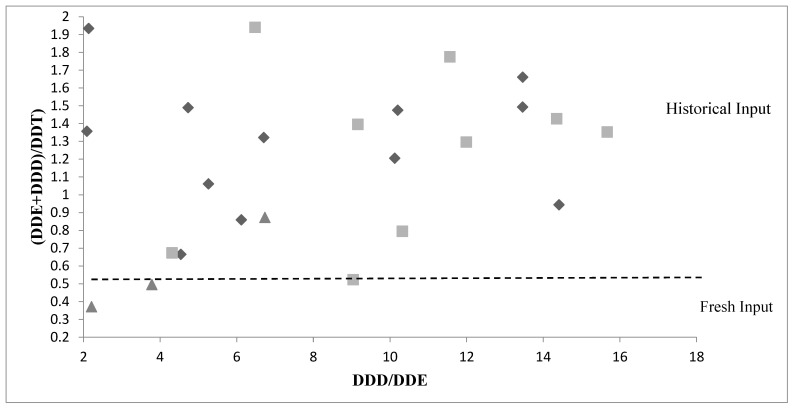
Isomeric ratios of DDT and its metabolites: DDD/DDE vs (DDE + DDD)/DDTs in the samples from Sele River. In the Figure the dotted line represents the point (0.5) where the fresh input becomes historical input. ◆ DP samples. ■ SPM samples. ▲ SED samples.

In this study, it was also significant to assess the biodegradation of DDT in its metabolites in the aquatic system. DDT not only controls crop pests and malaria but is also used as an active ingredient in antifouling coatings on fishing boats in several developing countries [52]; in Italy this pesticide has been prohibited from rural application and limited for public health [28]. DDT is composed of p,p′-DDT, p,p′-DDD, p,p′-DDE. DDT will dechlorinate to DDD under anaerobic conditions and degrade to DDE under aerobic conditions [52]. To determine the indicated levels of DDT in this study, the ratio of p,p′-DDT to its metabolites was estimated. When the ratio < 0.5, the DDT input was recent while when the ratio > 0.5 the DDT present in the environment is attributable to the historical input [53]. In the Sele river, the ratio in DP, SPM and sediment was 15.1, 16.6 and 18.7, respectively, so these data indicate that most of the DDTs in the Sele river were obtained from historical input (Figure 3).

Among the cyclodiene compounds and their metabolites, endosulfan sulfate was in abundance with the highest grades in water (DP + SPM), justifying 9% of total OCPs. Heptachlor epoxide is the metabolite of heptachlor and the ratio of heptachlor/heptachlor epoxide in the water system of the Sele river was 0.17. According to Kuranchie Mensah et al. [54], when the trend of metabolites was higher than the parent compound present, there were no fresh inputs of this contaminant in the water stream.

### 3.3. Spatiotemporal Diffusion

**Figure 4:**
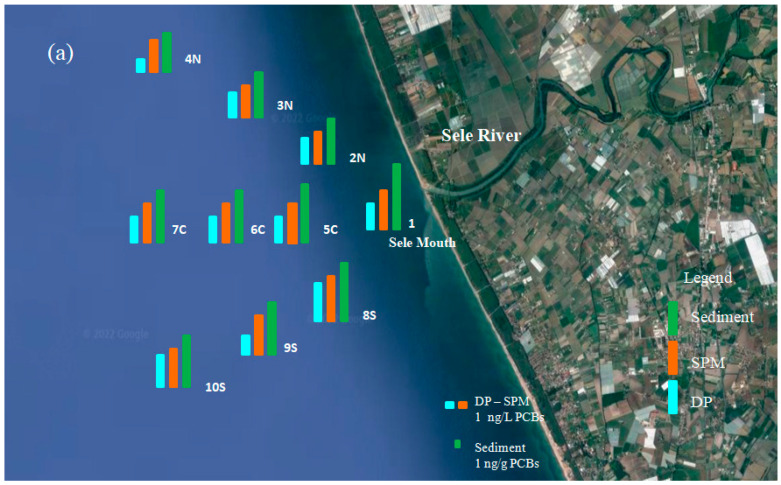

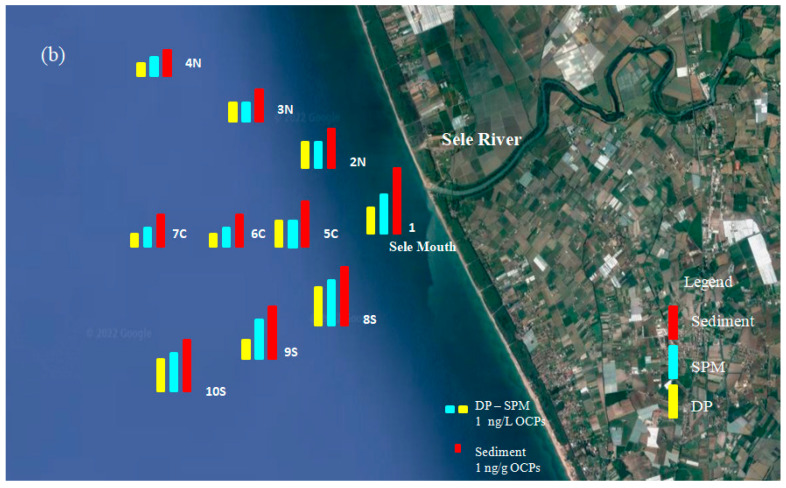
(**a**,**b**) show the spatial distribution of PCBs and OCPs in the water phase (DP ng/L), suspended particulate matter (SPM ng/L) and sediments (ng/g) from the Sele river.

The spatial diffusion designs of ∑ PCBs, ∑ OCPs and isomers concentrations in water and sediments of the Sele river are illustrated in Figure 4a,b, respectively. The results shown were obtained by studying and comparing the concentrations in the different sites in the dry and rainy seasons. The data showed a similar trend for both classes of compounds.

The Mouth of the Sele river is the most contaminated with a more elevated total concentration of PCBs and OCPs. Concentrations decrease as you move away from the mouth up to 1500 m from the coast, where the concentrations of PCBs and OCPs are significantly lower. Figure 4a,b show that the highest concentrations have been obtained around the Sele river mouth, as the contaminants present in the aqueous phase are diluted as one moves away. In particular, the contaminants load from the Sele river mouth has been shown to move southward into the Tyrrhenian Sea. In this study, the pollutant load drained into the Tyrrhenian Sea by the Sele river was also calculated. The results show that the total estimated value is equal to 89.7 kg year^−1^ (73.2 kg year^−1^ of PCBs and 16.5 kg year^−1^ of OCPs) In the water samples (DP phase), the total amount of pollutants was considerably lower mainly during the wet season (February), due to the abundant rains which caused water dilution effects. On the other hand, in SPM samples, the amounts were lowest in all sampling sites during the dry season. The results showed that the contaminants concentrations in DP decreased from July to February, in parallel with the increase in rainfall, which could cause dilution ratio variations. Therefore, the decrease of the pollutants amount moving from the Sele river mouth to the Mediterranean Sea is also affected by the high flow in the rainfall season, which results in an even higher dilution ratio. The lowest concentrations in SPM were recorded in the dry season (July), due to the decrease in flow and a greater stagnation of SPM, which led to the shift of contaminants from SPM to DP.

### 3.4. Potential Sources of PCBs

**Figure 5:**
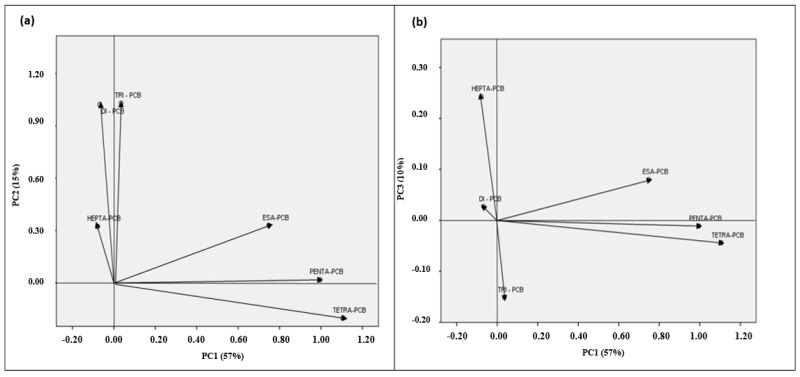
Principal Component Analysis (PCA) of the sediments PCBs results: (**a**) Score plot for the first and second principal component. (**b**) Loading plot for the first and third principal component.

For the purpose of more accurately controlling the emission and release of PCBs, it is deemed necessity to define their contamination sources as much as possible. Principal Component Analysis (PCA) has been executed on the different sediment datasets. Six groups of PCBs were identified in this study (Di- PCB, Tri-PCB, Tetra-PCB, Penta-PCB and Hepta-PCB). The obtained results from PCA manifested that the first three principal components show 57.1% (PC1), 15% (PC2) and 10% (PC3) of the total variance, respectively (Figure 5). Considering the three PCA axes individually, PC1 was principally composed of tetra-PCB, penta-PCB and hexa-PCB (high chlorinated congeners), PC2 was composed of Di-PCB and Tri-PCB, and the third component PC3 was composed of Hepta-PCBs.

The first component dominated by highly chlorinated PCBs could be unintentionally formed by anthropogenic activities such as industrial processes, waste incineration and vehicle exhaust [55,56]. Many studies [57,58] have demonstrated that PCB amount levels in the lower atmosphere near the water are confirmed 4Cl PCBs evaporated from the surface layer. In addition, the loss of a chlorine atom of highly chlorinated compounds with an anaerobic microbe can manifest in the sediments [26], which provides a good availability of molecules with few chlorine atoms. Therefore, PC1 represented PCBs originated from unintentionally formed local sources directly discharged into coastal water. The second component, dominated by 2Cl and 3Cl PCBs, suggests that these compounds could be transferred to the watercourse by surface runoff after rain cases, and cumulate in the estuary. The third component, composed of Hepta-PCB, suggests a point source deposition industrial loads along the Sele river: for example, discharge pipes from factories, sewage treatment plants and various organizations could be responsible for point source pollution in the Sele river. The existence can be assumed of a single major source in the watercourse related to the point source [59].

### 3.5. Dioxin Toxicity Equivalency

TEQs were calculated for eight PCBs (PCB 77, 105, 114, 118, 126, 156, 169 and 189) having dioxin-like properties by TEF, described in detail by Van den Berg et al. (2006) for all sediment samples. The TEQ concentrations of dioxin-like PCBs (DL-PCBs) detected at all sampling sites ranged from 0.004 to 0.270 ng/g with an average level of 0.050 ng/g. The highest ∑ TEQ_PCB_ concentrations were found at the Sele mouth (site 1). Despite PCB-114 indicating an amount higher than others PCB-DL, PCB-126 and PCB-169 contributed for 95.7% to TEQ_PCB_, because of their higher TEF.

The data indicated that TEQ_PCB_ values of the Sele river and its estuary were in a low level, suggesting that the toxicity of the PCBs in the watercourse could negatively cause a great threat to organisms and ecosystem, and endanger human health through bioconcentration and the food chain [38].

### 3.6. Risk Assessment of PCBs and OCPs

The SQGs guidelines can estimate the level of the possible negative effects and toxicity thresholds of specific organic contaminants in sediment for the ecological environment [60,61]. In this study, the total PCBs amount in sediment samples of the Sele river were considerably lower than PEL and ERM (Table 3), while 40% and 40% of analyzed samples indicated concentrations above TEL and ERL values, respectively, in the Sele river. and the risk factor of analyzed samples indicated concentrations above TEL and ERL values, respectively.

Regarding risk factors, the results showed that in the Sele river, the risk factor of PCBs for the sampling site were elevated at the mouth and at 500 m south, although in other sites, the risk value ranged from appreciable to low. Consequentially, based on the data obtained, the risk in the sediments of the Sele river was medium. Concerning the OCPs, in all analyzed samples, the ratio indicated a RQ < 1 for most of the pesticides. These data show that negative effects on the aquatic organism would rarely be observed [28,42,62,63].

## 4. Conclusions

This study analyzed the pollution characteristics, spatiotemporal variation, source identification and potential ecological risk of PCBs and OCPs in the Sele river; the input was also calculated of this watercourse into the Tyrrhenian Sea (Central Mediterranean Sea).

A higher amount of this contaminant was built in sediment samples than in their correspondent water bodies, DP and SPM, which suggests that suspension processes and sedimentation are principally in the Sele river. The data obtained showed that industrial procedure was reputed to be the principal source of PCBs; regarding the risk assessment, the risk factors of PCBs in sediment samples were elevated at the Sele river mouth and at 500 mt south, while in other sites, they are low. OCPs ratio, instead, was lower and showed an RQ < 1 for most analytes. Thus, the pollution situation in the Sele river and its estuary should be monitored regularly to assess the ecological risk in time. These data improve our knowledge on the Sele river water quality and they would inform such things as environmental monitoring, sediment quality guidelines application and ecological risk assessments. Our expectation is that the important and significative activity of establishing a rich database for different pollution factors can be developed, and more emerging contaminants should be included in ecological risk assessments of river ecosystems. Furthermore, this study’s results will help prevent future environmental water system contamination of the Sele river from PCBs and OCPs and strengthen prevention and pollution control measures against future risks. It would further help policymakers identify high-risk pollutants areas, improve environmental protection regulatory policy and sensitize the public to its importance. This study presents a novel result on the current status of water and sediment PCBs and OCPs levels in the area surrounding the Sele river. Therefore, the PCBs and OCPs levels in water and sediment from the Sele river should be further analyzed to ensure the contaminant levels reported in these areas are not being underestimated due to the continued increase in environmental activities.

## Figures and Tables

**Table 1:** Total PCBs concentrations in the three phases (DP, SPM, SED) analyzed from the samples collected from the Sele river, southern Italy.

Sampling Location			ΣPCBs								
Site Number Identificatin	Site	Sampling Point	DP (ng L^−1^)				SPM (ng L^−1^) (ng g^−1^ Dry wt)				SED (ng g^−1^ Dry wt)
			Apr	Jul	Nov	Feb	Apr	Jul	Nov	Feb	Apr
1 (river water)	Sele River Source	40°28′55″ N 14°56′33″ E	6.80	12.1	7.01	4.20	14.0 (1758.6)	9.21 (1026.2)	26.0 (2622.7)	35.1 (1895.3)	79.3
2 (sea water)	River Mouth at 500 mt North	40°29′04″ N 14°56′14″ E	5.71	6.70	6.56	4.68	2.11 (223.2)	2.85 (1236.0)	10.7 (402.2)	22.2 (179.0)	51.2
3 (sea water)	River Mouth at 500 mt Central	40°29′12″ N 14°55′56″ E	6.51	7.29	6.84	4.76	4.2 (1126.7)	5.04 (2514.3)	8.81 (2589.2)	6.85 (1674.7)	36.4
4 (sea water)	River Mouth at 500 mt South	40°29′20″ N 14°55′38″ E	8.72	10.2	7.77	5.02	7.00 (952.1)	6.18 (2698.2)	24.9 (589.5)	30.6 (212.5)	62.1
5 (sea water)	River Mouth at 1000 mt North	40°28′55″ N 14°56′12″ E	6.21	6.66	6.32	3.94	1.52 (118.0)	1.17 (263.1)	6.50 (374.3)	8.08 (125.3)	34.2
6 (sea water)	River Mouth at 1000 mt Central	40°28′55″ N 14°55′50″ E	6.35	5.90	6.71	3.74	2.90 (1569.3)	2.52 (1524.0)	3.40 (1348.7)	2.66 (910.3)	12.3
7 (sea water)	River Mouth at 1000 mt South	40°28′55″ N 14°55′28″ E	6.90	8.20	6.74	4.73	4.10 (460.5)	3.10 (325.3)	15.3 (548.6)	11.5 (84.2)	35.4
8 (sea water)	River Mouth at 1500 mt North	40°28′47″ N 14°56′16″ E	4.90	5.55	4.84	2.41	1.10 (106.9)	0.35 (36.4)	2.18 (774.0)	3.76 (1048.6)	19.2
9 (sea water)	River Mouth at 1500 mt Central	40°28′39″ N 14°55′56″ E	5.30	5.89	5.00	1.98	3.22 (582.2)	1.00 (614.3)	1.50 (486.1)	1.78 (547.2)	5.0
10 (sea water)	River Mouth at 1500 mt South	40°28′30″ N 14°55′38″ E	7.00	7.22	4.32	3.16	4.21 (1986.5)	2.12 (156.1)	4.10 (120.3)	7.54 (1486.4)	10.1

**Table 2:** Total OCPs concentrations in the three phases (DP, SPM, SED) analyzed for the samples collected from the Sele river, southern Italy.

Sampling Location			ΣOCPs								
Site Number Identification	Site	Sampling Point	DP (ng L^−1^)				SPM (ng L^−1^) (ng g^−1^ Dry wt)				SED (ng g^−1^ Dry wt)
			Apr	Jul	Nov	Feb	Apr	Jul	Nov	Feb	Apr
1 (river water)	Sele River Source	40°48′54.03″ N 14°36′45.36″ E	4.01	5.71	3.75	1.95	2.08 (198.5)	1.56 (154.1)	3.98 (243.0)	4.82 (201.4)	15.2
2 (sea water)	River Mouth at 500 mt North	40°46′42.73″ N 14°34′00.48″ E	1.70	2.98	2.12	1.10	1.06 (70.2	0.55 (284.1)	1.22 (51.8)	1.80 (174.3)	1.39
3 (sea water)	River Mouth at 500 mt Central	40°46′00.34″ N 14°33′10.68″ E	2.03	2.01	1.98	0.80	1.20 (185.4)	0.50 (154.2)	1.26 (274.6)	1.86 (119.4)	1.54
4 (sea water)	River Mouth at 500 mt South	40°43′42.62″ N 14°28′07.89″ E	3.24	4.38	2.18	1.82	1.48 (150.2)	0.68 (298.4)	1.54 (97.5)	2.20 (241.2)	3.85
5 (sea water)	River Mouth at 1000 mt North	40°43′40.11″ N 14°28′06.45″ E	1.00	2.00	1.78	0.75	1.00 (94.1)	0.48 (102.3)	0.98 (95.4)	1.03 (100.1)	1.20
6 (sea water)	River Mouth at 1000 mt Central	40°43′42.46″ N 14°28′05.03″ E	0.98	1.32	1.20	0.49	1.10 (254.3)	0.32 (36.8)	0.99 (198.4)	1.23 (155.2)	1.32
7 (sea water)	River Mouth at 1000 mt South	40°43′45.09″ N 14°28′05.17″ E	2.12	2.85	1.60	1.10	1.24 (110.4)	0.38 (89.2)	1.26 (114.7)	1.30 (10.2)	2.84
8 (sea water)	River Mouth at 1500 mt North	40°43′35.68″ N 14°28′02.94″ E	0.84	1.20	0.90	0.70	0.50 (71.4)	0.39 (96.8)	0.84 (195.7)	1.00 (180.3)	1.21
9 (sea water)	River Mouth at 1500 mt Central	40°43′42.25″ N 14°27′59.97″ E	0.84	0.90	0.81	0.36	0.47 (112.2)	0.05 (65.3)	0.91 (117.8)	0.89 (148.3)	1.10
10 (sea water)	River Mouth at 1500 mt South	40°43′49.26″ N 14°27′59.82″ E	1.45	1.85	0.73	0.79	0.60 (196.5)	0.10 (52.7)	0.74 (17.4)	0.89 (185.6)	1.02

**Table 3:** A comparison of the TEL, PEL, ERL and ERM guideline values (µg Kg^−1^) for PCBs and OCPs and data from the Sele river and estuary, southern Italy.

	TEL	Percentage over the TEL	PEL	Percentage over the PEL	ERL	Percentage over the ERL	ERM	Percentage over the ERM
**PCBs**								
Total PCBs	21.6	40	189	0	22.7	40	180	0
**OCPs**								
γ-HCH (lindane)	0.32	0	0.99	0	-		-	
Dieldrin	0.72	0	4.3	0	0.02	50	8	0
4,4-DDD	1.22	20	7.81	0	2	0	20	0
4,4-DDE	2.07	0	374	0	2.2	0	27	0
4,4-DDT	1.19	10	4.77	0	1	10	7	0
Total DDT	3.89	0	51.7	0	1.58	10	46.1	0

## Data Availability

The datasets obtained and analyzed in the current study are available from the corresponding author on reasonable request.

## Supplementary Materials

The following supporting information can be downloaded at: https://www.mdpi.com/article/10.3390/toxics10110662/s1, Table S1: Percentage of PCBs in the different seasons in the water samples (DP) from Sele River (Southern Italy); Table S2: Percentage of PCBs in the different seasons in the suspended particulate matter (SPM) from Sele River (Southern Italy), Table S3: Percentage of PCBs in the April month in the sediment samples (SED) from Sele River (Southern Italy); Table S4: Environmental data from sampling sites: Table S5: Percentage of OCPs in the different seasons in the water samples (DP) from Sele River (Southern Italy); Table S6: Percentage of OCPs in the different seasons in the suspended particulate matter (SPM) from Sele River (Southern Italy); Table S7: Percentage of OCPs in the April month in the sediment samples (SED) from Sele River (Southern Italy); Table S8: Description of concentration of PCBs in water dissolved phase (DP) samples from Sele River, (Southern Italy); Table S9: Description of concentration of PCBs in Suspended Particulate Matter (SPM) samples from Sele River, (Southern Italy): Table S10: Description of concentration of PCBs in Sediment (SED) samples from Sele River, (Southern Italy); Table S11: Description of concentration of OCPs in water dissolved phase (DP) samples from Sele River, (Southern Italy); Table S12: Description of concentration of OCPs in Suspended Particulate Matter (SPM) samples from Sele River, (Southern Italy); Table S13: Description of concentration of OCPs in Sediment (SED) samples from Sele River, (Southern Italy).
