# Supplementary material for: Occurrence and Distribution of Persistent Organic Pollutants (POPs) from Sele River, Southern Italy: Analysis of Polychlorinated Biphenyls and Organochlorine Pesticides in a Water–Sediment System
Source: Toxics. 2022 Nov 4;10(11):662. doi: 10.3390/toxics10110662 (PMC9697341; doi:10.3390/toxics10110662)
Supplement: Supplementary file 1 [file toxics-10-00662-s001.zip › toxics-1991177-supplementary.pdf]

Supplementary Materials: Occurrence and distribution of persistent organic pollutants (POPs) from Sele River southern Italy: Analysis of polychlorinated biphenyls and organochlorine pesticides in a water-sediment system

Elvira De Rosa. Paolo Montuori. Maria Triassi. Armando Masucci and Antonio Nardone

Table S1. Percentage of PCBs in the different seasons in the water samples (DP) from Sele River (Southern Italy).

					% PCBs		
Season	Matrix	Site	DI+TRI	TETRA	PENTA	ESA	EPTA
NOV	DP	River Mouth	7.3	31.7	23.0	24.2	13.7
NOV	DP	Nord 500mt	7.7	31.7	18.8	33.4	8.4
NOV	DP	Nord 1000mt	7.6	42.9	23.2	22.4	3.9
NOV	DP	Nord 1500mt	6.7	38.5	18.6	26.8	9.3
NOV	DP	Central 500mt	7.6	42.6	21.2	23.6	5.0
NOV	DP	Central 1000mt	6.9	38.2	25.1	24.4	5.5
NOV	DP	Central 1500mt	6.8	35.7	21.8	26.5	9.2
NOV	DP	South 500mt	7.9	36.1	29.2	26.6	0.3
NOV	DP	South 1000mt	7.6	34.9	26.9	21.9	8.8
NOV	DP	South 1500mt	8.5	38.9	26.5	24.2	2.0
FEB	DP	River Mouth	6.4	36.7	15.0	27.6	14.4
FEB	DP	Nord 500mt	6.3	37.5	14.5	28.2	13.5
FEB	DP	Nord 1000mt	5.6	30.0	18.6	32.1	13.6
FEB	DP	Nord 1500mt	0.0	55.2	15.0	13.1	16.7
FEB	DP	Central 500mt	7.6	39.7	15.3	26.9	10.5
FEB	DP	Central 1000mt	4.7	44.1	10.4	32.6	8.2
FEB	DP	Central 1500mt	0.2	37.3	18.1	34.1	10.4
FEB	DP	South 500mt	6.9	37.7	17.9	27.6	9.8
FEB	DP	South 1000mt	7.9	40.0	15.4	27.8	8.9
FEB	DP	South 1500mt	8.0	39.7	15.9	29.2	7.2
APR	DP	River Mouth	6.9	30.2	22.6	24.4	15.8
APR	DP	Nord 500mt	19.8	28.4	18.2	28.4	5.2
APR	DP	Nord 1000mt	8.2	45.9	22.3	19.6	4.0
APR	DP	Nord 1500mt	6.8	36.8	21.3	26.8	8.3
APR	DP	Central 500mt	7.5	43.1	20.1	23.2	6.1
APR	DP	Central 1000mt	7.0	38.1	23.8	25.4	5.7
APR	DP	Central 1500mt	5.0	38.0	25.2	23.9	8.0
APR	DP	South 500mt	8.0	37.7	33.7	20.0	0.6
APR	DP	South 1000mt	7.0	34.3	28.6	22.5	7.6
APR	DP	South 1500mt	4.3	36.0	30.4	20.2	9.1
JUL	DP	River Mouth	11.2	29.4	21.7	23.0	14.7
JUL	DP	Nord 500mt	8.3	37.3	33.4	20.9	0.1
JUL	DP	Nord 1000mt	4.0	36.3	33.0	19.5	7.2
JUL	DP	Nord 1500mt	19.9	32.8	23.2	17.3	6.9
JUL	DP	Central 500mt	8.7	39.3	30.0	21.6	0.4
JUL	DP	Central 1000mt	6.8	33.6	29.6	21.2	8.7
JUL	DP	Central 1500mt	4.7	33.9	31.2	22.6	7.7
JUL	DP	South 500mt	18.9	32.6	31.3	16.8	0.4
JUL	DP	South 1000mt	6.2	38.8	26.9	21.1	7.1
JUL	DP	South 1500mt	4.9	34.4	31.8	20.4	8.5

Table S2. Percentage of PCBs in the different seasons in the suspended particulate matter (SPM) from Sele River (Southern Italy).

			% PCBs				
Season	Matrix	Site	DI+TRI	TETRA	PENTA	ESA	EPTA
NOV	SPM	River Mouth	7.3	32.1	37.6	16.5	6.5
NOV	SPM	Nord 500mt	6.8	35.1	31.2	19.4	7.4
NOV	SPM	Nord 1000mt	7.1	34.1	26.0	24.2	8.6
NOV	SPM	Nord 1500mt	4.3	36.6	30.3	22.6	6.3
NOV	SPM	Central 500mt	7.8	39.3	27.2	20.9	4.7
NOV	SPM	Central 1000mt	7.7	38.5	18.0	28.3	7.4
NOV	SPM	Central 1500mt	6.5	36.4	17.5	30.2	9.5
NOV	SPM	South 500mt	8.0	36.5	32.4	18.6	4.5
NOV	SPM	South 1000mt	8.0	37.5	30.6	19.4	4.6
NOV	SPM	South 1500mt	6.9	30.2	32.3	23.4	7.2
FEB	SPM	River Mouth	7.4	31.5	38.8	15.9	6.3
FEB	SPM	Nord 500mt	8.0	36.8	31.8	18.8	4.6
FEB	SPM	Nord 1000mt	7.2	33.3	28.3	23.0	8.2
FEB	SPM	Nord 1500mt	13.6	31.8	22.6	18.7	13.4
FEB	SPM	Central 500mt	12.4	27.9	33.1	15.6	11.0
FEB	SPM	Central 1000mt	8.3	37.4	22.3	24.3	7.7
FEB	SPM	Central 1500mt	10.5	13.3	60.6	5.9	9.8
FEB	SPM	South 500mt	7.7	31.6	33.9	19.5	7.4
FEB	SPM	South 1000mt	8.3	35.4	26.6	22.2	7.5
FEB	SPM	South 1500mt	8.4	37.3	23.4	24.2	6.8
APR	SPM	River Mouth	7.3	28.7	29.4	21.1	13.5
APR	SPM	Nord 500mt	8.7	29.3	38.0	19.5	4.5
APR	SPM	Nord 1000mt	10.6	13.1	53.9	14.5	7.9
APR	SPM	Nord 1500mt	11.2	15.7	18.5	42.2	12.4
APR	SPM	Central 500mt	7.5	44.6	7.6	16.4	23.8
APR	SPM	Central 1000mt	20.7	33.8	15.1	18.8	11.6
APR	SPM	Central 1500mt	0.5	49.8	17.1	27.6	4.9
APR	SPM	South 500mt	7.4	33.7	32.1	17.6	9.3
APR	SPM	South 1000mt	4.6	45.4	24.0	18.7	7.2
APR	SPM	South 1500mt	2.0	44.8	23.8	19.9	9.6
JUL	SPM	River Mouth	11.1	29.1	23.2	22.3	14.3
JUL	SPM	Nord 500mt	1.3	30.5	18.4	25.2	24.7
JUL	SPM	Nord 1000mt	5.0	35.1	19.2	11.6	29.0
JUL	SPM	Nord 1500mt	7.2	41.5	26.4	19.6	5.4
JUL	SPM	Central 500mt	4.0	35.8	32.5	20.5	7.2
JUL	SPM	Central 1000mt	6.0	32.8	27.1	19.2	14.9
JUL	SPM	Central 1500mt	5.1	40.2	19.9	26.8	8.0
JUL	SPM	South 500mt	9.1	37.2	26.6	20.6	6.4
JUL	SPM	South 1000mt	9.2	38.8	23.2	22.1	6.8
JUL	SPM	South 1500mt	0.0	8.4	65.3	14.0	12.3

Table S3. Percentage of PCBs in the April month in the sediment samples (SED) from Sele River (Southern Italy).

			% PCBs				
Season	Matrix	Site	DI+TRI	TETRA	PENTA	ESA	EPTA
APR	SED	River Mouth	5.1	20.0	31.0	25.7	18.2
APR	SED	Nord 500mt	0.8	32.2	39.2	23.2	4.5
APR	SED	Nord 1000mt	4.8	18.7	29.0	24.2	23.3
APR	SED	Nord 1500mt	7.9	27.8	36.4	17.9	10.0
APR	SED	Central 500mt	0.8	32.2	39.2	23.3	4.5
APR	SED	Central 1000mt	1.1	23.0	32.6	25.3	18.1
APR	SED	Central 1500mt	1.0	23.0	32.1	25.6	18.4
APR	SED	South 500mt	0.9	32.2	39.3	23.2	4.5
APR	SED	South 1000mt	0.9	32.6	39.8	22.4	4.3
APR	SED	South 1500mt	4.6	18.8	29.0	24.2	23.3

Table S4. Environmental data from sampling sites.

**Site Number Identification**	**Sampling Site**	pH				Temperature (°C)				Salinity (‰)			
		Apr	Jul	Nov	Feb	Apr	Jul	Nov	Feb	Apr	Jul	Nov	Feb
1 (river water)	Sele River Source	7.1	7.8	7.0	7.5	20.2	25.0	19.5	15.1	9.9	7.4	8.6	7.9
2 (sea water)	River Mouth at 500mt North	7.0	7.21	7.3	7.2	22.4	23.9	17.6	15.3	10.7	11.3	7.8	10.7
3 (sea water)	River Mouth at 500mt Central	7.4	7.5	7.8	7.2	20.6	23.5	19.2	14.5	10.7	9.3	6.6	10.0
4 (sea water)	River Mouth at 500mt South	7.0	7.5	7.0	7.5	19.8	24.3	18.0	14.9	8.3	9.0	5.2	7.8
5 (sea water)	River Mouth at 1000mt North	6.7	7.4	7.1	7.3	21.4	22.9	18.3	18.2	13.2	13.7	13.4	12.9
6 (sea water)	River Mouth at 1000mt Central	7.8	7.3	7.5	7.2	18.9	24.7	19.0	15.5	18.4	12.1	15.2	11.7
7 (sea water)	River Mouth at 1000mt South	7.7	7.2	7.2	7.6	22.4	23.2	17.9	17.6	14.9	16.0	15.5	15.7
8 (sea water)	River Mouth at 1500mt North	7.2	7.5	7.1	6.8	21.4	24.9	18.8	15.4	19.2	22.7	23.6	23.8
9 (sea water)	River Mouth at 1500mt Central	7.2	7.8	7.9	7.3	22.1	25.1	19.6	14.9	21.4	22.7	22.1	22.6
10 (sea water)	River Mouth at 1500mt South	7.5	7.6	7.2	7.3	21.1	23.	18.5	15.4	21.2	22.2	20.8	21.6

Table S5. Percentage of OCPs in the different seasons in the water samples (DP) from Sele River (Southern Italy).

			% OCPs		
Season	Matrix	Site	HCH	DDT	CYCLO
NOV	DP	River Mouth	21.0	18.2	60.7
NOV	DP	Nord 500mt	31.5	14.7	53.8
NOV	DP	Nord 1000mt	32.7	14.7	52.6
NOV	DP	Nord 1500mt	31.2	12.8	56.1
NOV	DP	Central 500mt	19.9	18.0	62.1
NOV	DP	Central 1000mt	31.6	9.4	59.0
NOV	DP	Central 1500mt	36.2	0.0	63.8
NOV	DP	South 500mt	34.7	16.2	49.2
NOV	DP	South 1000mt	58.1	0.0	41.9
NOV	DP	South 1500mt	63.3	16.2	20.6
FEB	DP	River Mouth	0.4	29.8	69.8
FEB	DP	Nord 500mt	45.2	22.6	32.2
FEB	DP	Nord 1000mt	9.9	33.9	56.2
FEB	DP	Nord 1500mt	4.2	71.5	24.2
FEB	DP	Central 500mt	6.5	45.1	48.4
FEB	DP	Central 1000mt	11.5	55.2	33.3
FEB	DP	Central 1500mt	0.0	34.0	66.0
FEB	DP	South 500mt	39.2	37.0	23.8
FEB	DP	South 1000mt	31.2	30.7	38.1
FEB	DP	South 1500mt	42.3	32.0	25.7
APR	DP	River Mouth	0.0	26.8	73.2
APR	DP	Nord 500mt	20.1	17.1	62.8
APR	DP	Nord 1000mt	30.3	25.9	43.7
APR	DP	Nord 1500mt	63.5	0.0	36.5
APR	DP	Central 500mt	36.0	22.2	41.8
APR	DP	Central 1000mt	32.1	23.5	44.3
APR	DP	Central 1500mt	45.5	11.9	42.6
APR	DP	South 500mt	26.7	39.1	34.2
APR	DP	South 1000mt	33.3	21.9	44.8
APR	DP	South 1500mt	34.9	23.5	41.7
JUL	DP	River Mouth	0.1	22.5	77.5
JUL	DP	Nord 500mt	29.2	29.3	41.5
JUL	DP	Nord 1000mt	25.5	33.8	40.6
JUL	DP	Nord 1500mt	29.8	24.2	45.9
JUL	DP	Central 500mt	39.1	14.8	46.1
JUL	DP	Central 1000mt	3.5	66.7	29.8
JUL	DP	Central 1500mt	16.5	58.7	24.8
JUL	DP	South 500mt	26.9	15.3	57.8
JUL	DP	South 1000mt	33.2	14.7	52.1
JUL	DP	South 1500mt	32.7	25.2	42.1

Table S6. Percentage of OCPs in the different seasons in the suspended particulate matter (SPM) from Sele River (Southern Italy).

			% OCPs		
Season	Matrix	Site	HCH	DDT	CYCLO
NOV	SPM	River Mouth	19.0	22.6	58.4
NOV	SPM	Nord 500mt	41.3	14.8	43.9
NOV	SPM	Nord 1000mt	33.3	7.8	58.9
NOV	SPM	Nord 1500mt	37.5	12.0	50.5
NOV	SPM	Central 500mt	33.8	26.1	40.1
NOV	SPM	Central 1000mt	35.0	9.8	55.2
NOV	SPM	Central 1500mt	65.2	0.0	34.8
NOV	SPM	South 500mt	39.8	14.5	45.8
NOV	SPM	South 1000mt	45.2	0.0	54.8
NOV	SPM	South 1500mt	56.8	15.0	28.2
FEB	SPM	River Mouth	16.9	16.9	66.2
FEB	SPM	Nord 500mt	33.8	16.5	49.6
FEB	SPM	Nord 1000mt	10.0	32.7	57.3
FEB	SPM	Nord 1500mt	9.8	45.0	45.1
FEB	SPM	Central 500mt	8.8	41.6	49.6
FEB	SPM	Central 1000mt	11.3	57.6	31.1
FEB	SPM	Central 1500mt	0.0	32.8	67.2
FEB	SPM	South 500mt	35.9	21.1	43.0
FEB	SPM	South 1000mt	31.7	32.5	35.8
FEB	SPM	South 1500mt	51.4	22.1	26.5
APR	SPM	River Mouth	40.3	13.7	46.0
APR	SPM	Nord 500mt	50.0	29.1	20.9
APR	SPM	Nord 1000mt	60.6	30.1	9.3
APR	SPM	Nord 1500mt	76.3	0.0	23.7
APR	SPM	Central 500mt	35.1	23.7	41.2
APR	SPM	Central 1000mt	40.8	10.9	48.3
APR	SPM	Central 1500mt	71.8	18.7	9.5
APR	SPM	South 500mt	40.1	14.4	45.5
APR	SPM	South 1000mt	34.4	24.6	41.0
APR	SPM	South 1500mt	9.1	24.3	66.6
JUL	SPM	River Mouth	37.6	17.1	45.3
JUL	SPM	Nord 500mt	49.3	0.4	50.3
JUL	SPM	Nord 1000mt	49.8	0.4	49.8
JUL	SPM	Nord 1500mt	81.8	0.7	17.5
JUL	SPM	Central 500mt	67.1	27.3	5.6
JUL	SPM	Central 1000mt	4.9	83.9	11.2
JUL	SPM	Central 1500mt	6.8	87.7	5.5
JUL	SPM	South 500mt	39.3	40.3	20.4
JUL	SPM	South 1000mt	1.8	98.2	0.0
JUL	SPM	South 1500mt	5.3	94.7	0.0

Table S7. Percentage of OCPs in the April month in the sediment samples (SED) from Sele River (Southern Italy).

			% OCPs		
Season	Matrix	Site	HCH	DDT	CYCLO
APR	SED	River Mouth	13.6	52.8	33.6
APR	SED	Nord 500mt	5.2	30.3	64.5
APR	SED	Nord 1000mt	4.8	31.2	64.0
APR	SED	Nord 1500mt	4.5	8.6	86.8
APR	SED	Central 500mt	6.6	30.9	62.5
APR	SED	Central 1000mt	4.8	83.3	11.9
APR	SED	Central 1500mt	16.6	22.5	60.9
APR	SED	South 500mt	28.9	34.1	37.0
APR	SED	South 1000mt	2.7	79.5	17.7
APR	SED	South 1500mt	6.6	8.6	84.9

Table S8. Description of concentration of PCBs in water dissolved phase (DP) samples from Sele River. (Southern Italy)

	8	28	37	44	49	52	60	66	70	74	77	82	87	99	101	105	114	118	126	128	138	153	156	158	166	169	170	179	180	183	187	189
**November**																																
River Mouth	0.76	0.21	0.02	0.82	0.46	0.14	0.64	0.10	0.59	0.18	0.32	0.01	0.08	0.08	0.06	0.02	0.07	0.27	0.04	ND	0.10	0.09	0.15	0.48	0.30	0.01	0.43	0.09	0.13	0.03	0.29	0.02
Nord at 500mt	0.37	0.09	0.05	0.33	0.10	0.12	0.05	0.62	0.60	0.20	0.06	0.16	0.07	0.11	0.14	0.02	0.20	0.29	0.24	0.47	0.05	0.27	0.52	0.48	0.31	0.09	0.22	0.09	0.09	0.06	0.03	0.07
Nord at 1000mt	0.39	0.01	0.07	0.40	0.19	0.13	0.38	0.85	0.35	0.01	0.06	0.06	0.09	0.04	0.30	ND	0.67	0.31	0.22	0.57	ND	0.26	ND	0.45	0.17	0.09	0.11	0.09	0.04	0.01	0.01	0.02
Nord at 1500mt	0.31	ND	0.02	0.32	0.42	0.12	0.30	0.09	0.54	0.05	0.03	0.26	0.07	0.04	0.02	0.01	0.20	0.24	0.07	0.25	0.14	0.26	0.02	0.39	0.15	0.08	0.13	0.01	0.08	0.01	0.17	0.04
Central at 500mt	0.34	0.08	0.02	0.32	0.10	0.13	0.05	0.68	0.55	0.65	0.02	0.17	0.07	0.03	0.56	0.01	0.20	0.23	0.07	0.23	0.08	0.30	0.01	0.13	0.49	0.05	0.09	0.02	0.04	0.02	1.02	0.07
Central at 1000mt	0.39	0.01	0.07	0.40	0.19	0.13	0.38	0.85	0.35	0.01	0.06	0.06	0.09	0.04	0.30	ND	0.67	0.31	0.22	0.57	ND	0.26	ND	0.45	0.17	0.09	0.51	0.06	0.04	0.01	0.03	0.02
Central at 1500mt	0.30	0.02	0.02	0.31	0.41	0.12	0.30	0.09	0.53	ND	0.03	0.36	0.07	0.07	0.02	ND	0.21	0.26	0.10	0.27	0.12	0.25	0.03	0.39	0.16	0.11	0.13	0.01	0.08	0.01	0.17	0.06
South at 500mt	0.35	0.12	0.08	0.31	0.47	0.13	0.34	0.11	0.58	0.19	0.41	0.41	0.08	0.12	0.15	ND	0.58	0.28	0.43	0.42	0.17	0.34	0.11	0.44	0.28	0.11	0.39	0.09	0.10	0.03	0.12	0.08
South at 1000mt	0.32	0.12	0.07	0.30	0.44	0.12	0.38	0.10	0.60	0.07	0.35	0.30	0.07	0.06	0.07	ND	0.64	0.27	0.41	0.40	0.16	0.20	0.10	0.35	0.18	0.10	0.26	0.08	0.09	0.02	0.07	0.08
South at 1500mt	0.30	0.07	0.06	0.27	0.42	0.11	0.04	0.10	0.05	0.03	0.07	0.07	0.07	0.01	ND	ND	0.50	0.24	0.37	0.30	0.10	0.08	0.08	0.33	0.06	0.09	0.25	0.04	0.03	0.02	0.09	0.07
**February**																																
River Mouth	0.09	0.05	0.02	0.03	0.42	0.01	0.49	0.99	0.56	0.05	0.07	0.13	0.07	0.03	0.01	ND	0.09	ND	0.01	0.06	0.05	0.31	0.04	0.02	0.15	ND	0.06	0.02	0.08	0.10	0.09	0.10
Nord at 500mt	0.28	ND	0.02	0.29	0.39	0.11	0.30	0.09	0.53	0.03	0.02	0.05	0.06	0.01	0.03	ND	0.20	0.24	0.08	0.25	0.12	0.26	0.05	0.39	0.16	0.09	0.27	0.02	0.08	0.03	0.17	0.05
Nord at 1000mt	0.20	ND	0.02	0.27	0.24	0.10	0.19	0.09	0.23	ND	0.06	0.01	0.07	0.02	0.14	ND	0.20	0.24	0.07	0.25	0.09	0.27	0.04	0.38	0.16	0.08	0.21	0.02	0.08	0.03	0.16	0.03
Nord at 1500mt	ND	ND	ND	0.04	0.23	0.12	0.30	ND	0.55	0.03	0.06	0.02	0.07	ND	0.02	ND	ND	0.25	ND	ND	0.05	ND	0.04	ND	0.14	0.08	0.09	ND	0.08	0.02	0.16	0.04
Central at 500mt	0.32	0.03	0.02	0.30	0.38	0.11	0.34	0.10	0.56	0.05	0.06	0.04	0.07	0.04	0.03	0.01	0.21	0.25	0.08	0.26	0.07	0.28	0.03	0.40	0.16	0.06	0.18	0.02	0.08	0.02	0.16	0.02
Central at 1000mt	0.17	ND	ND	0.28	0.37	0.10	0.30	ND	0.52	0.03	0.06	0.03	0.07	0.03	0.02	ND	ND	0.25	ND	0.25	0.06	0.16	0.03	0.39	0.16	0.07	0.09	ND	0.08	0.01	0.08	0.04
Central at 1500mt	ND	0.01	ND	0.16	0.22	0.10	0.29	ND	ND	0.01	0.08	0.07	0.06	0.04	ND	ND	ND	0.14	ND	0.15	0.07	0.11	ND	0.05	0.12	0.08	0.11	ND	0.07	0.01	ND	0.04
South at 500mt	0.80	0.03	0.02	0.29	0.37	0.11	0.30	0.10	0.58	0.07	0.08	0.07	0.08	0.03	0.08	ND	0.20	0.26	0.08	0.28	0.16	0.30	0.02	0.09	0.16	0.08	0.13	0.02	0.08	0.06	0.08	0.02
South at 1000mt	0.31	0.05	0.02	0.29	0.37	0.11	0.33	0.10	0.60	0.04	0.06	0.03	0.07	0.07	ND	0.01	0.21	0.26	0.09	0.26	0.05	0.30	0.03	0.42	0.18	0.07	0.13	0.02	0.08	0.02	0.13	0.04
South at 1500mt	0.30	0.02	ND	0.08	0.02	0.11	0.29	ND	0.52	ND	ND	0.02	0.06	0.02	0.01	ND	0.20	0.24	0.07	0.24	0.06	0.06	0.02	0.24	0.15	0.08	0.09	ND	0.08	0.06	0.07	0.04
**April**																																
River Mouth	0.38	0.66	0.69	0.02	0.14	0.13	0.23	0.10	0.57	0.15	0.18	1.29	0.07	0.08	0.04	0.02	0.08	0.08	0.45	0.05	0.19	0.28	0.03	0.07	0.27	0.02	0.06	0.09	0.14	0.08	0.05	0.12
Nord at 500mt	0.09	0.08	0.06	0.06	0.10	0.13	0.05	0.86	0.64	0.17	0.09	0.07	0.07	0.01	0.15	0.02	0.20	0.26	0.33	0.50	0.07	0.27	0.56	0.09	0.30	0.09	0.09	0.08	0.08	0.05	0.03	0.06
Nord at 1000mt	0.40	0.09	0.02	0.35	0.10	0.13	0.34	0.74	0.62	0.51	0.06	0.15	0.07	0.03	0.59	0.02	0.21	0.25	0.07	0.23	0.09	0.32	0.02	0.13	0.37	0.05	0.11	0.02	0.04	0.02	0.02	0.05
Nord at 1500mt	0.10	0.03	0.02	0.34	0.38	0.11	0.33	0.09	0.54	0.02	0.06	0.14	0.06	0.02	0.34	ND	0.20	0.24	0.07	0.25	0.16	0.29	0.02	0.39	0.18	0.09	0.11	0.01	0.08	0.02	0.17	0.04
Central at 500mt	0.39	0.06	0.06	0.38	0.43	0.13	0.36	0.71	0.71	0.18	0.13	0.08	0.05	0.03	0.14	ND	0.57	0.27	0.17	0.49	0.03	0.28	0.02	0.40	0.27	0.03	0.01	0.02	0.02	0.02	0.03	0.04
Central at 1000mt	0.35	0.03	0.06	1.34	0.39	0.12	0.32	0.74	0.37	0.07	0.08	0.14	0.08	0.06	0.13	ND	0.01	0.26	0.23	0.43	0.02	0.36	0.07	0.04	0.18	0.05	0.10	0.08	0.09	0.02	0.08	0.04
Central at 1500mt	0.20	0.03	0.02	0.22	0.42	0.12	0.31	0.10	0.59	0.05	0.07	0.38	0.07	0.04	0.15	0.01	0.23	0.26	0.09	0.12	0.51	0.24	0.03	0.41	0.16	0.10	0.09	0.02	0.06	0.01	0.14	0.06
South at 500mt	0.37	0.12	0.08	0.32	0.49	0.13	0.36	0.51	0.61	0.23	0.40	0.60	0.08	0.16	0.17	ND	0.61	0.29	0.46	0.31	0.16	0.07	0.11	0.40	0.25	0.10	0.46	0.09	0.10	0.02	0.60	0.08
South at 1000mt	0.34	0.08	0.06	0.32	0.39	0.12	0.37	0.10	0.99	0.12	0.35	0.07	0.08	0.10	0.16	0.01	0.68	0.27	0.41	0.41	0.13	0.05	0.10	0.39	0.19	0.09	0.22	0.06	0.08	0.02	0.07	0.07
South at 1500mt	0.18	0.01	0.07	0.35	0.45	0.11	0.29	0.12	0.61	ND	0.12	0.68	0.07	0.02	0.25	0.01	0.63	0.26	0.39	0.25	0.18	0.17	0.08	0.37	0.15	0.09	0.21	0.07	0.09	0.32	0.18	0.21
**July**																																
River Mouth	1.57	0.40	0.15	1.58	0.48	0.14	0.35	0.13	0.64	0.21	0.43	0.42	0.08	0.16	0.03	0.09	0.62	0.28	0.68	0.73	0.25	0.02	0.17	0.59	0.32	0.12	0.67	0.15	0.13	0.06	0.22	0.17
Nord at 500mt	0.36	0.12	0.10	0.32	0.48	0.13	0.35	0.10	0.09	0.23	0.39	0.59	0.08	0.16	0.17	ND	0.59	0.28	0.35	0.21	0.16	0.07	0.11	0.09	0.32	0.10	0.35	0.08	0.11	0.02	0.14	0.08
Nord at 1000mt	0.15	0.06	0.07	0.36	0.43	0.12	0.34	0.11	0.65	0.13	0.37	0.36	0.07	0.17	0.15	0.01	0.72	0.30	0.38	0.28	0.14	0.18	0.06	0.43	0.15	0.06	0.15	0.07	0.07	0.02	0.06	0.05
Nord at 1500mt	0.20	0.03	0.06	0.34	0.48	0.12	0.32	0.12	0.63	0.05	0.09	0.07	0.06	0.03	0.17	ND	0.57	0.24	0.37	0.26	0.06	0.15	0.06	0.35	0.16	0.09	0.17	0.07	0.08	0.01	0.07	0.05
Central at 500mt	0.33	0.08	0.07	0.19	0.39	0.12	0.32	0.90	0.54	0.17	0.20	0.25	0.07	0.10	0.05	ND	0.55	0.27	0.20	0.26	0.10	0.08	0.10	0.39	0.21	0.05	0.31	0.08	0.79	0.01	0.02	0.07
Central at 1000mt	0.31	0.04	0.06	0.30	0.38	0.12	0.61	0.79	0.26	0.05	0.06	0.07	0.07	0.04	0.16	0.01	0.67	0.25	0.45	0.26	0.11	0.15	0.06	0.03	0.06	0.09	0.20	0.05	0.01	0.02	0.07	0.06
Central at 1500mt	0.18	0.03	0.07	0.20	0.46	0.12	0.33	0.19	0.23	0.08	0.09	0.06	0.07	0.02	0.36	0.01	0.62	0.26	0.45	0.30	0.08	0.20	0.09	0.38	0.19	0.09	0.15	0.07	0.09	0.02	0.12	0.30
South at 500mt	0.43	0.29	0.09	0.36	0.17	0.14	0.59	0.18	0.72	0.28	0.48	0.75	0.10	0.30	0.37	0.01	0.64	0.32	0.51	0.29	0.18	0.16	0.11	0.43	0.34	0.40	0.39	0.09	0.12	0.72	0.12	0.19
South at 1000mt	0.37	0.11	0.07	0.38	0.47	0.69	0.43	0.14	0.02	0.17	0.02	0.34	0.09	0.14	0.27	0.01	0.74	0.31	0.49	0.48	0.16	0.29	0.11	0.47	0.26	0.10	0.26	0.28	0.10	0.08	0.29	0.08
South at 1500mt	0.04	0.04	0.08	0.41	0.33	0.14	0.36	0.14	0.09	0.15	0.06	0.32	0.05	0.03	0.41	0.02	0.72	0.31	0.02	0.91	0.12	0.14	0.09	0.42	0.24	0.10	0.26	0.01	1.10	0.02	ND	0.10

ND: No Detectable

Table S9. Description of concentration of PCBs in Suspended Particulate Matter (SPM) samples from Sele River. (Southern Italy)

	**8**	**28**	**37**	**44**	**49**	**52**	**60**	**66**	**70**	**74**	**77**	**82**	**87**	**99**	**101**	**105**	**114**	**118**	**126**	**128**	**138**	**153**	**156**	**158**	**166**	**169**	**170**	**179**	**180**	**183**	**187**	**189**
**November**																																
River Mouth	0.94	0.18	0.23	0.07	1.26	0.25	0.99	2.00	0.77	1.85	1.17	3.88	0.19	1.27	0.37	0.14	0.35	0.75	2.18	1.05	0.77	1.08	0.20	1.10	0.84	0.25	0.72	0.13	0.20	0.13	0.51	0.20
Nord at 500mt	0.39	0.17	0.10	0.41	0.52	0.13	0.39	0.79	0.69	0.23	0.17	0.80	0.08	0.22	0.43	0.02	0.23	0.32	0.85	0.37	0.20	0.35	0.05	0.48	0.24	0.13	0.20	0.06	0.05	0.03	0.24	1.38
Nord at 1000mt	0.34	0.10	0.02	0.36	0.46	0.12	0.34	0.10	0.61	0.13	0.11	0.60	0.07	0.15	0.25	0.01	0.22	0.29	0.11	0.32	0.16	0.30	0.04	0.43	0.20	0.12	0.17	0.01	0.09	0.02	0.21	0.07
Nord at 1500mt	ND	0.12	ND	0.01	0.27	ND	ND	0.11	ND	0.19	0.21	ND	0.08	0.15	ND	ND	0.12	0.20	0.06	0.21	0.12	ND	ND	ND	0.08	0.09	ND	ND	0.02	0.03	ND	0.04
Central at 500mt	0.01	0.26	0.08	0.51	0.56	0.14	0.48	0.95	0.75	0.54	0.34	0.61	0.09	0.27	0.73	0.03	0.65	0.31	0.22	0.20	0.11	0.10	0.03	0.10	0.12	0.10	0.17	0.12	0.10	0.04	0.03	0.05
Central at 1000mt	0.31	0.02	ND	0.29	0.04	0.12	0.31	ND	0.14	ND	0.05	0.02	0.07	0.02	0.01	ND	ND	0.25	0.41	0.25	0.06	0.27	0.03	0.36	0.16	0.04	0.09	0.02	0.04	0.01	0.07	0.04
Central at 1500mt	0.10	0.01	ND	0.13	0.19	0.09	ND	ND	0.25	ND	ND	ND	0.05	ND	ND	ND	ND	0.10	0.06	0.02	0.02	0.06	ND	0.22	0.06	0.07	0.06	0.01	0.02	ND	ND	0.03
South at 500mt	1.03	0.98	0.15	1.05	1.10	0.24	0.98	1.94	1.53	1.87	1.13	2.33	0.17	0.97	2.84	0.11	0.09	0.60	0.43	1.49	0.38	0.93	0.10	0.96	0.19	0.17	0.47	0.24	0.18	0.13	0.11	0.11
South at 1000mt	0.72	0.16	0.11	0.72	0.77	0.18	0.68	1.35	1.07	1.09	0.67	1.32	0.12	0.56	1.60	0.06	0.63	0.03	0.30	1.02	0.22	0.01	0.06	0.68	0.05	0.13	0.29	0.17	0.10	0.06	0.08	0.27
South at 1500mt	0.19	0.14	0.02	0.05	0.29	0.14	ND	0.11	0.53	ND	0.21	0.35	0.08	0.13	0.03	0.02	0.22	0.31	0.09	0.19	0.11	0.16	0.06	0.37	ND	0.02	0.16	ND	0.10	0.01	ND	0.03
**February**																																
River Mouth	1.71	0.55	0.42	0.97	0.28	0.41	1.82	0.66	3.24	4.03	2.51	8.04	0.34	0.68	1.33	0.30	0.51	0.03	1.01	0.97	1.54	ND	0.40	0.96	0.05	0.16	0.02	0.24	0.35	0.26	1.05	0.27
Nord at 500mt	0.87	0.78	0.13	0.88	0.93	0.21	0.83	1.64	1.30	1.50	0.91	1.85	0.15	0.77	2.25	0.08	1.10	0.51	0.36	1.25	0.30	0.77	0.08	0.81	0.82	0.14	0.38	0.20	0.16	0.10	0.08	0.09
Nord at 1000mt	0.39	0.18	0.02	0.42	0.52	0.13	0.39	0.11	0.69	0.24	0.18	0.82	0.08	0.23	0.46	0.02	0.23	0.33	0.12	0.38	0.21	0.36	0.06	0.48	0.25	0.13	0.20	0.02	0.10	0.03	0.24	0.08
Nord at 1500mt	0.39	0.11	0.01	0.15	0.21	0.10	0.27	0.14	0.30	ND	0.03	0.49	0.06	0.06	ND	0.01	ND	0.23	ND	0.14	0.05	0.10	ND	0.24	0.10	0.08	0.09	0.02	0.08	0.01	0.28	0.03
Central at 500mt	0.54	0.29	0.02	0.21	0.27	0.11	0.37	0.18	0.41	0.16	0.19	1.04	0.08	0.22	0.20	0.03	0.28	0.30	0.13	0.21	0.11	0.16	ND	0.31	0.18	0.09	0.16	0.02	0.10	0.03	0.41	0.04
Central at 1000mt	0.16	0.06	ND	0.15	0.21	0.10	0.26	ND	0.29	ND	ND	ND	0.03	ND	ND	ND	0.24	0.22	0.10	0.13	0.03	0.08	ND	0.24	0.08	0.08	0.08	0.02	0.08	0.01	ND	0.03
Central at 1500mt	ND	0.19	ND	0.24	ND	ND	ND	ND	ND	ND	ND	0.54	ND	0.22	ND	ND	ND	0.33	ND	ND	ND	ND	0.01	ND	ND	0.10	ND	0.02	0.10	ND	ND	0.05
South at 500mt	1.02	1.30	0.04	1.06	1.19	0.25	1.07	0.27	2.10	2.29	1.47	3.51	0.24	1.24	3.93	0.14	0.33	0.80	0.19	1.16	0.98	1.38	0.16	1.20	0.91	0.19	0.78	0.04	0.22	0.26	0.77	0.19
South at 1000mt	0.52	0.42	0.02	0.51	0.61	0.15	0.58	0.15	1.04	0.62	0.42	0.84	0.11	0.43	0.84	0.05	0.25	0.42	0.13	0.49	0.19	0.60	0.07	0.68	0.41	0.12	0.31	0.03	0.12	0.07	0.27	0.07
South at 1500mt	0.42	0.21	ND	0.41	0.45	0.14	0.42	0.11	0.75	0.28	0.26	0.47	0.09	0.19	0.57	0.03	ND	0.32	0.09	0.37	0.15	0.42	0.05	0.46	0.27	0.10	0.18	0.02	0.10	0.03	0.12	0.05
**April**																																
River Mouth	0.62	0.59	0.02	0.54	0.72	0.18	0.56	0.14	0.96	0.80	0.97	0.27	0.12	0.43	0.04	0.07	0.89	0.44	0.75	0.80	0.43	0.55	0.33	0.74	0.55	0.18	0.10	0.15	0.22	0.08	0.55	0.20
Nord at 500mt	ND	0.24	ND	0.25	0.11	0.12	ND	0.12	ND	ND	ND	ND	0.09	0.17	ND	0.03	0.22	0.14	0.09	ND	0.17	ND	0.01	ND	0.05	0.08	ND	0.02	ND	0.04	0.02	0.15
Nord at 1000mt	ND	0.18	ND	ND	ND	0.12	ND	0.11	ND	ND	ND	ND	0.09	0.22	ND	0.02	0.02	0.32	0.08	ND	0.15	ND	0.01	ND	ND	0.10	ND	0.02	ND	0.06	ND	0.06
Nord at 1500mt	0.11	0.06	ND	ND	ND	0.10	ND	0.14	ND	ND	ND	ND	0.06	ND	ND	ND	ND	0.12	ND	0.13	0.03	0.08	ND	0.04	0.08	0.08	0.07	ND	0.04	0.01	ND	0.03
Central at 500mt	0.40	0.02	0.06	0.38	0.44	0.13	0.37	0.72	0.62	0.13	0.10	ND	ND	ND	0.04	ND	ND	0.28	0.18	0.02	0.02	0.17	0.02	ND	0.13	ND	ND	0.02	ND	ND	ND	ND
Central at 1000mt	0.24	0.17	ND	0.17	0.23	0.10	0.30	0.09	0.33	0.01	0.08	0.08	0.07	0.04	ND	ND	0.10	0.05	ND	0.16	0.07	0.02	ND	0.26	0.32	ND	0.02	0.04	ND	ND	0.01	ND
Central at 1500mt	ND	0.01	ND	0.17	0.23	0.10	0.31	0.09	0.33	0.02	0.09	0.10	0.07	0.05	ND	ND	ND	0.25	ND	0.16	0.07	0.12	ND	0.27	0.13	ND	0.12	0.02	0.51	ND	ND	ND
South at 500mt	0.39	0.17	0.08	0.05	0.52	0.14	0.38	0.11	0.65	0.32	0.08	0.75	0.09	0.21	0.31	0.01	0.64	0.31	0.49	0.34	0.19	0.08	0.12	0.02	0.08	0.10	0.02	0.09	0.10	0.03	0.11	0.01
South at 1000mt	ND	0.27	ND	0.26	0.32	0.12	0.06	0.12	0.10	0.45	0.38	ND	ND	ND	0.09	0.03	0.22	0.35	0.09	0.18	0.19	0.24	0.01	ND	0.26	0.10	0.14	0.02	0.10	0.04	ND	ND
South at 1500mt	ND	0.07	ND	0.19	0.24	0.10	0.33	ND	0.36	0.11	0.15	0.23	0.07	0.10	0.11	0.01	ND	0.27	ND	0.18	0.09	0.14	ND	ND	0.15	0.08	0.14	ND	0.09	0.04	0.95	0.05
**July**																																
River Mouth	0.11	0.48	0.17	0.62	0.51	0.04	0.37	0.13	0.08	0.31	0.51	0.07	0.08	0.22	0.18	0.10	0.16	0.30	0.52	0.58	0.29	0.35	0.19	0.63	0.36	0.13	0.73	0.16	0.14	0.07	0.33	0.29
Nord at 500mt	ND	0.05	ND	0.18	ND	0.10	0.32	ND	0.35	ND	0.13	0.18	0.07	0.08	0.06	0.01	ND	0.26	ND	0.18	0.09	0.13	ND	0.27	0.04	0.08	0.13	ND	0.08	0.02	ND	0.05
Nord at 1000mt	ND	0.14	0.02	0.17	ND	0.10	0.09	0.06	0.02	ND	0.06	0.02	0.07	0.02	ND	ND	0.05	0.04	ND	ND	0.04	0.11	ND	ND	0.01	0.16	ND	ND	ND	ND	ND	ND
Nord at 1500mt	ND	0.20	ND	0.08	ND	ND	ND	ND	ND	0.06	ND	ND	ND	ND	ND	ND	ND	ND	ND	ND	ND	ND	ND	ND	ND	ND	ND	ND	ND	ND	ND	ND
Central at 500mt	ND	0.31	ND	0.29	0.05	0.13	ND	ND	0.05	0.52	0.43	0.85	0.10	0.34	0.80	0.04	ND	0.32	ND	0.30	0.21	0.06	0.02	0.09	0.01	0.01	0.07	ND	0.02	0.05	0.01	ND
Central at 1000mt	ND	ND	ND	0.05	ND	0.07	0.05	ND	0.08	ND	ND	0.89	0.10	ND	0.85	ND	ND	0.09	ND	0.10	ND	ND	0.02	ND	ND	0.09	ND	ND	0.04	0.05	0.01	0.06
Central at 1500mt	ND	0.12	0.02	0.16	0.02	0.10	0.28	ND	0.01	ND	0.05	ND	0.06	0.01	ND	ND	0.04	0.04	ND	0.05	0.05	ND	ND	0.05	0.01	0.08	0.10	ND	0.08	0.01	ND	0.04
South at 500mt	0.15	0.32	0.02	0.22	0.27	0.11	0.38	0.19	0.41	0.19	0.21	0.35	0.08	0.15	0.24	0.01	0.28	0.30	ND	0.22	0.12	0.17	ND	0.31	0.19	0.09	0.17	ND	0.10	0.03	0.75	0.05
South at 1000mt	0.14	0.03	0.02	0.19	0.25	0.11	0.04	0.18	0.37	0.09	0.04	0.20	0.07	0.09	0.08	0.01	0.17	0.27	ND	0.19	0.09	0.04	ND	0.09	0.15	0.09	0.04	ND	0.09	0.02	ND	0.01
South at 1500mt	ND	ND	ND	0.05	ND	0.07	0.05	ND	0.08	ND	ND	0.09	0.10	ND	0.55	ND	ND	0.09	ND	0.10	ND	ND	0.02	ND	ND	0.09	ND	0.42	0.04	0.05	0.22	0.09

ND: No Detectable

Table S10. Description of concentration of PCBs in Sediment (SED) samples from Sele River. (Southern Italy)

	**8**	**28**	**37**	**44**	**49**	**52**	**60**	**66**	**70**	**74**	**77**	**82**	**87**	**99**	**101**	**105**	**114**	**118**	**126**	**128**	**138**	**153**	**156**	**158**	**166**	**169**	**170**	**179**	**180**	**183**	**187**	**189**
**April**																																
River Mouth	1.66	1.54	0.11	2.31	2.45	0.26	0.74	2.95	1.34	1.24	1.59	0.26	0.25	2.33	8.70	0.45	2.25	3.43	2.27	2.53	1.66	3.32	0.12	2.25	6.92	3.75	1.05	7.28	1.54	4.70	3.48	4.65
Nord at 500mt	0.08	0.14	0.03	1.55	0.17	0.36	0.25	2.91	3.77	0.24	0.16	0.95	0.31	3.86	0.55	0.04	2.74	2.82	0.19	1.59	1.39	0.10	0.26	2.41	1.03	0.01	0.11	0.30	0.01	5.01	8.61	9.25
Nord at 1000mt	0.54	0.49	0.04	0.75	0.79	0.08	0.24	0.96	0.43	0.39	0.51	0.07	0.08	0.75	2.79	0.15	0.73	1.11	0.74	0.82	0.54	1.07	0.04	0.73	0.94	1.21	0.44	0.08	1.18	2.91	8.48	4.18
Nord at 1500mt	0.23	0.49	0.01	0.23	0.29	0.05	0.24	0.01	0.41	0.70	0.64	1.04	0.04	0.38	1.07	0.06	0.31	0.15	0.30	0.32	0.26	0.28	0.14	0.28	0.31	0.05	0.40	0.06	0.07	0.05	1.25	9.19
Central at 500mt	0.05	0.09	0.02	0.98	0.11	0.23	0.16	1.84	2.39	0.14	0.10	0.59	0.20	2.44	2.34	0.03	1.73	1.79	0.12	1.01	0.88	0.06	0.17	1.52	0.65	0.01	0.07	0.19	0.01	0.01	7.39	9.16
Central at 1000mt	0.02	0.04	0.05	0.47	0.05	0.61	0.07	0.48	0.62	0.03	0.04	0.05	0.53	0.73	0.01	0.07	0.60	1.32	0.05	0.46	0.61	0.02	0.47	0.70	0.31	0.03	0.05	0.72	0.02	0.02	0.47	2.59
Central at 1500mt	0.01	0.01	0.02	0.20	0.02	0.26	0.03	0.21	0.27	ND	0.01	ND	0.23	0.31	ND	0.03	0.26	0.57	0.02	0.20	0.26	0.01	0.20	0.30	0.13	0.01	0.02	0.11	0.01	0.01	0.20	1.16
South at 500mt	0.13	0.26	0.04	2.71	0.30	0.63	0.43	5.08	6.60	0.43	0.29	1.69	0.54	6.76	0.99	0.08	4.79	4.93	0.33	2.78	2.43	0.17	0.46	4.21	1.80	0.02	0.20	0.53	1.03	1.02	1.17	9.24
South at 1000mt	0.08	0.16	0.03	1.66	0.18	0.38	0.26	3.10	4.03	0.26	0.17	1.02	0.33	4.12	0.59	0.05	2.92	3.01	0.20	1.70	1.48	0.10	0.28	2.02	1.10	0.01	0.13	0.33	0.02	0.02	0.65	5.08
South at 1500mt	0.63	0.23	0.04	0.18	0.23	0.10	0.28	0.12	0.11	0.46	0.60	0.05	0.10	0.88	0.29	0.17	0.86	0.31	0.26	0.96	0.63	0.16	0.05	0.76	0.11	0.12	0.20	0.09	0.21	0.41	0.16	0.39

ND: No Detectable

Table S11. Description of concentration of OCPs in water dissolved phase (DP) samples from Sele River. (Southern Italy)

	Lindane	a-HCH	b-HCH	d- HCH	Heptacloro	Aldrin	H.epossido	a-endos.	b-endos.	E. Sulfate	DDE	DDD	DDT	Metox	Dieldrin	Endrin
**November**																
River Mouth	0.36	0.09	0.29	0.06	0.06	0.01	0.34	0.79	0.21	0.32	0.27	0.26	0.05	0.10	0.45	0.11
Nord at 500mt	0.30	0.06	0.30	0.01	0.02	0.06	0.22	0.15	0.07	0.25	0.14	0.11	0.02	0.03	0.34	0.02
Nord at 1000mt	0.29	0.06	0.29	0.01	0.02	0.04	0.19	0.13	0.06	0.14	0.14	0.11	0.04	0.03	0.22	0.02
Nord at 1500mt	ND	ND	0.28	0.01	0.01	ND	0.07	0.04	0.06	0.24	ND	0.09	ND	0.02	ND	0.08
Central at 500mt	ND	ND	0.35	0.04	0.05	ND	0.21	0.14	0.07	0.34	0.21	0.11	0.01	0.05	0.40	0.01
Central at 1000mt	ND	ND	0.35	0.04	0.05	ND	0.19	0.06	0.13	0.25	ND	0.10	ND	0.02	ND	0.01
Central at 1500mt	ND	ND	0.32	ND	ND	ND	0.17	0.10	ND	0.23	ND	ND	ND	ND	ND	ND
South at 500mt	0.38	0.06	0.30	0.04	0.06	ND	0.21	0.14	0.07	0.26	0.15	0.11	0.06	0.04	0.15	0.14
South at 1000mt	ND	0.06	0.33	ND	ND	ND	0.19	1.03	ND	ND	ND	ND	ND	ND	ND	ND
South at 1500mt	ND	0.06	0.27	0.01	ND	ND	0.07	0.04	ND	ND	ND	0.29	ND	ND	ND	ND
**February**																
River Mouth	0.35	0.06	0.28	0.01	0.02	ND	0.08	0.18	0.07	0.23	0.16	0.11	0.06	0.03	0.30	0.02
Nord at 500mt	0.29	ND	0.29	0.01	0.01	0.06	0.19	0.10	0.07	ND	0.04	0.02	0.01	0.03	ND	ND
Nord at 1000mt	ND	0.09	ND	0.01	0.01	ND	0.18	0.04	0.07	0.05	0.10	0.11	ND	0.08	ND	0.01
Nord at 1500mt	ND	0.06	ND	ND	0.05	ND	0.18	0.10	ND	ND	0.21	0.09	ND	0.02	ND	0.01
Central at 500mt	ND	0.06	ND	ND	0.06	0.07	0.19	0.09	ND	ND	0.10	0.21	0.01	ND	ND	0.01
Central at 1000mt	ND	0.06	ND	ND	0.02	ND	0.09	0.04	ND	ND	0.06	0.10	0.01	0.09	ND	0.01
Central at 1500mt	ND	ND	ND	ND	0.02	ND	0.17	0.09	ND	ND	0.04	ND	0.01	ND	ND	0.02
South at 500mt	0.38	0.12	0.30	0.10	0.02	0.01	0.09	0.14	0.24	ND	0.28	ND	0.05	0.03	ND	0.07
South at 1000mt	0.36	ND	ND	ND	0.01	ND	0.31	0.12	ND	ND	0.21	0.03	0.06	ND	ND	ND
South at 1500mt	0.34	ND	ND	ND	0.01	ND	0.08	0.12	ND	ND	0.14	0.10	0.01	ND	ND	ND
**April**																
River Mouth	0.55	0.09	0.32	0.04	0.09	0.51	0.39	0.58	0.11	0.26	0.13	0.10	0.38	0.08	0.37	0.07
Nord at 500mt	ND	ND	0.31	0.01	0.01	ND	0.19	0.10	0.07	0.26	0.11	0.10	0.01	ND	0.31	0.21
Nord at 1000mt	ND	ND	0.29	ND	0.01	ND	0.07	0.05	0.06	0.24	0.15	0.05	0.09	ND	ND	ND
Nord at 1500mt	ND	0.06	0.32	ND	ND	ND	0.13	0.10	ND	ND	ND	ND	ND	0.24	ND	ND
Central at 500mt	ND	0.06	0.28	0.01	ND	ND	0.07	0.04	0.06	0.23	0.14	0.02	ND	ND	1.14	ND
Central at 1000mt	ND	ND	0.28	0.01	ND	ND	0.06	0.04	0.06	0.23	0.23	0.07	ND	ND	ND	ND
Central at 1500mt	ND	ND	0.27	0.01	ND	ND	ND	0.04	ND	0.22	ND	0.07	ND	ND	ND	0.23
South at 500mt	0.28	0.05	0.27	0.01	0.01	0.06	0.06	0.05	0.07	1.03	0.16	0.08	ND	1.01	0.11	ND
South at 1000mt	ND	0.05	0.29	ND	ND	0.06	0.07	0.04	0.06	0.23	0.13	0.08	1.00	0.11	ND	ND
South at 1500mt	ND	0.05	0.17	ND	ND	ND	0.07	0.03	0.05	0.02	ND	0.04	ND	1.01	ND	ND
**July**																
River Mouth	0.47	0.14	0.29	0.09	0.17	0.13	0.66	1.07	0.36	0.46	0.31	0.51	0.14	0.22	0.52	0.18
Nord at 500mt	0.30	0.06	0.29	0.01	0.05	ND	0.07	0.10	0.13	0.24	0.28	0.10	0.01	0.02	1.32	0.01
Nord at 1000mt	ND	ND	0.27	0.01	0.02	ND	ND	0.04	0.06	ND	0.26	0.07	0.01	0.02	0.31	1.02
Nord at 1500mt	ND	ND	0.18	0.01	0.01	ND	ND	ND	0.02	ND	0.01	0.02	ND	0.01	1.03	ND
Central at 500mt	0.28	0.05	0.27	ND	0.01	0.01	0.05	0.04	0.06	0.12	0.05	0.01	ND	0.01	0.01	1.00
Central at 1000mt	0.28	ND	0.27	0.01	0.01	ND	ND	ND	0.06	ND	0.10	0.06	ND	0.01	0.51	ND
Central at 1500mt	ND	ND	0.27	0.01	0.01	ND	ND	0.04	0.05	ND	0.14	0.05	ND	0.01	0.31	0.01
South at 500mt	0.28	0.11	0.27	0.01	0.02	0.18	0.06	0.20	0.07	0.41	1.27	0.09	0.01	0.01	0.30	1.10
South at 1000mt	0.29	0.10	0.27	0.01	0.01	0.05	0.07	0.13	0.06	0.35	0.19	0.08	0.01	0.82	0.32	0.10
South at 1500mt	ND	0.06	0.30	0.01	ND	ND	ND	0.05	0.07	ND	0.16	0.10	0.01	0.75	0.36	ND

ND: No Detectable

Table S12**.** Description of concentration of OCPs in Suspended Particulate Matter (SPM) samples from Sele River. (Southern Italy)

	Lindane	a-HCH	b-HCH	d- HCH	Heptacloro	Aldrin	H.epossido	a-endos.	b-endos.	E. Sulfate	DDE	DDD	DDT	Metox	Dieldrin	Endrin
**November**																
River Mouth	0.35	0.09	0.08	0.05	0.06	ND	0.03	0.75	0.19	0.07	0.27	0.43	0.11	0.27	0.43	0.80
Nord at 500mt	0.09	0.06	0.28	0.01	ND	ND	0.07	0.04	ND	0.23	0.03	0.08	ND	0.01	0.30	0.02
Nord at 1000mt	ND	0.05	0.27	0.01	ND	ND	ND	0.04	ND	0.22	ND	0.06	ND	ND	0.31	ND
Nord at 1500mt	ND	ND	0.27	0.01	ND	ND	0.05	0.04	0.05	0.23	ND	0.07	ND	0.12	ND	ND
Central at 500mt	ND	ND	0.28	0.01	0.01	ND	ND	0.24	0.16	0.24	0.23	0.07	ND	0.01	ND	0.01
Central at 1000mt	ND	ND	0.27	ND	ND	ND	0.15	ND	0.16	ND	ND	0.07	ND	ND	0.33	ND
Central at 1500mt	ND	ND	0.29	ND	ND	0.16	0.06	0.04	0.06	ND	ND	0.15	ND	ND	0.16	ND
South at 500mt	0.31	0.06	0.29	0.01	0.01	ND	0.07	0.05	0.06	0.24	0.14	0.08	0.01	0.01	0.13	0.07
South at 1000mt	ND	0.06	0.28	0.01	0.21	ND	0.07	0.04	0.06	0.23	ND	ND	ND	ND	0.30	ND
South at 1500mt	ND	0.05	0.27	0.01	ND	ND	0.07	0.04	0.05	ND	ND	0.07	ND	0.01	0.16	ND
**February**																
River Mouth	0.38	0.09	0.44	0.06	0.06	0.29	0.43	0.77	0.20	0.41	0.26	0.41	0.10	0.32	0.41	0.20
Nord at 500mt	0.31	ND	0.29	0.01	0.01	0.06	0.07	0.10	0.07	0.25	0.15	0.11	0.01	0.02	0.33	0.01
Nord at 1000mt	ND	0.09	ND	0.01	0.01	ND	0.19	0.05	0.07	0.24	0.14	0.10	ND	0.08	ND	0.05
Nord at 1500mt	ND	0.06	ND	ND	0.01	ND	0.20	0.04	0.15	ND	0.14	0.10	ND	0.05	0.15	0.11
Central at 500mt	ND	0.06	ND	ND	0.03	0.05	0.22	0.04	ND	0.12	0.15	0.10	0.21	0.22	0.26	0.41
Central at 1000mt	ND	0.06	ND	ND	0.01	ND	0.09	0.05	ND	0.25	0.15	0.11	0.01	0.22	0.25	0.04
Central at 1500mt	ND	ND	ND	ND	0.01	ND	0.20	0.10	ND	0.18	0.15	ND	0.01	0.20	ND	0.04
South at 500mt	0.37	0.12	0.28	0.07	0.01	0.01	0.08	0.12	0.19	0.23	0.16	0.09	0.06	0.05	0.31	0.04
South at 1000mt	0.31	ND	ND	ND	0.01	ND	0.20	0.11	ND	ND	0.21	0.10	0.21	ND	0.14	ND
South at 1500mt	0.34	ND	ND	ND	0.01	ND	0.07	0.09	ND	ND	0.17	ND	0.21	ND	ND	ND
**April**																
River Mouth	0.48	0.06	0.30	0.01	0.01	ND	0.08	0.05	0.07	0.26	0.15	0.11	0.01	0.02	0.45	0.01
Nord at 500mt	ND	ND	0.28	0.19	ND	ND	0.07	0.04	0.15	0.16	0.15	ND	ND	0.02	ND	ND
Nord at 1000mt	ND	ND	0.29	ND	ND	ND	ND	0.04	ND	0.16	0.14	ND	ND	0.11	0.25	ND
Nord at 1500mt	ND	0.05	0.28	ND	ND	ND	0.09	0.09	ND	ND	ND	ND	ND	ND	ND	ND
Central at 500mt	ND	0.05	0.27	ND	ND	ND	0.06	0.04	0.06	0.22	0.13	0.07	0.01	0.01	0.26	ND
Central at 1000mt	ND	ND	0.27	0.01	ND	ND	0.06	0.04	ND	0.23	ND	0.07	ND	ND	0.28	0.14
Central at 1500mt	ND	ND	0.29	0.01	ND	ND	ND	0.09	ND	ND	ND	0.09	ND	ND	ND	ND
South at 500mt	0.28	0.05	0.27	0.01	ND	ND	0.05	0.04	0.06	0.12	0.13	0.07	0.06	0.01	0.21	0.10
South at 1000mt	ND	0.06	0.29	ND	ND	ND	0.07	0.04	0.06	0.24	0.15	0.08	0.01	0.24	ND	ND
South at 1500mt	ND	0.05	ND	ND	ND	ND	0.07	0.04	0.05	0.23	0.13	ND	ND	0.03	ND	ND
**July**																
River Mouth	0.30	0.06	0.28	0.01	0.01	ND	0.08	0.05	0.07	0.23	0.05	0.11	0.01	0.03	0.26	0.01
Nord at 500mt	0.29	ND	ND	ND	ND	ND	ND	ND	0.04	0.22	ND	ND	ND	ND	ND	ND
Nord at 1000mt	0.28	ND	ND	ND	ND	ND	ND	ND	0.05	0.12	ND	ND	0.03	ND	ND	ND
Nord at 1500mt	0.29	ND	ND	ND	ND	ND	ND	ND	0.06	ND	ND	ND	0.03	ND	ND	ND
Central at 500mt	ND	ND	0.27	ND	0.01	0.01	ND	ND	ND	ND	ND	0.07	0.01	0.02	0.09	ND
Central at 1000mt	ND	ND	ND	0.01	0.01	ND	ND	ND	ND	ND	ND	0.03	ND	0.01	0.13	0.13
Central at 1500mt	ND	ND	ND	0.01	ND	ND	ND	ND	ND	ND	ND	0.04	ND	ND	ND	ND
South at 500mt	0.28	ND	ND	0.01	ND	ND	ND	0.15	ND	ND	0.08	0.09	0.08	ND	ND	ND
South at 1000mt	ND	ND	ND	0.01	ND	ND	ND	ND	ND	ND	0.20	0.09	0.09	ND	ND	ND
South at 1500mt	ND	ND	ND	0.01	ND	ND	ND	ND	ND	ND	ND	0.07	0.02	ND	ND	ND

ND: No Detectable

Table S13**.** Description of concentration of OCPs in Sediment (SED) samples from Sele River. (Southern Italy)

	Lindane	a-HCH	b-HCH	d- HCH	Heptacloro	Aldrin	H.epossido	a-endos.	b-endos.	E. Sulfate	DDE	DDD	DDT	Metox	Dieldrin	Endrin
**April**																
River Mouth	0.75	0.15	0.26	0.18	0.27	0.02	2.03	0.33	1.45	0.05	1.37	2.93	1.12	1.00	1.16	2.21
Nord at 500mt	0.03	0.04	0.07	0.05	0.07	0.03	0.01	0.08	0.01	0.18	0.35	0.24	0.03	0.05	0.07	0.09
Nord at 1000mt	0.02	0.02	0.01	0.02	0.05	0.06	0.46	0.06	0.04	0.09	0.17	0.08	0.01	0.06	0.04	0.02
Nord at 1500mt	0.02	0.01	0.01	0.01	0.02	0.03	0.60	0.03	0.02	0.30	0.02	0.05	0.01	0.03	0.03	0.01
Central at 500mt	0.03	0.02	0.03	0.02	0.02	0.03	0.63	0.03	0.05	0.03	0.02	0.08	0.30	0.02	0.22	0.01
Central at 1000mt	0.02	0.01	0.01	0.01	ND	0.01	0.01	0.02	0.03	0.02	0.02	0.74	0.01	0.16	0.02	0.22
Central at 1500mt	0.02	0.03	0.04	ND	0.02	0.03	0.03	0.04	0.02	0.02	0.71	0.02	0.03	0.02	0.03	0.01
South at 500mt	0.03	0.04	0.07	0.05	0.07	1.03	0.51	0.08	0.11	0.38	0.35	0.24	0.03	0.45	0.17	0.24
South at 1000mt	0.03	0.02	0.01	0.01	ND	0.01	0.01	0.02	0.03	0.03	0.12	1.26	0.01	0.41	0.61	0.27
South at 1500mt	0.03	0.02	0.04	ND	0.02	0.04	0.06	0.12	0.12	0.04	0.05	0.01	0.02	0.03	0.20	0.21

ND: No Detectable
